# Globin Digest Improves Visceral Adiposity Through UCP1 Upregulation in Diet-Induced Obese Zebrafish and Mice

**DOI:** 10.3389/fnut.2021.650975

**Published:** 2021-09-27

**Authors:** Liqing Zang, Yasuhito Shimada, Hiroko Nakayama, Izumi Matsuoka, Youngil Kim, Djong-Chi Chu, Lekh Raj Juneja, Rika Tsuruta, Yuka Sasakawa, Junya Kuroyanagi, Norihiro Nishimura

**Affiliations:** ^1^Graduate School of Regional Innovation Studies, Mie University, Tsu, Japan; ^2^Mie University Zebrafish Drug Screening Center, Tsu, Japan; ^3^Department of Integrative Pharmacology, Mie University Graduate School of Medicine, Tsu, Japan; ^4^Department of Bioinformatics, Mie University Advanced Science Research Promotion Center, Tsu, Japan; ^5^Rohto Pharmaceutical Co., Ltd., Osaka, Japan; ^6^MG Pharma Inc., Osaka, Japan; ^7^UMOU Science Lab, Matsusaka, Japan

**Keywords:** metabolic syndrome, natural product, comparative transcriptomics, oligopeptides, zebrafish, obesity

## Abstract

Globin digest (GD), a bioactive oligopeptide derived from porcine hemoglobin proteins, has been demonstrated to have beneficial effects on improving postprandial hyperlipidemia, hyperglycemia, and liver injury. We previously reported the lipid-lowering effects of GD using a zebrafish obesogenic test. Here, we sought to evaluate the effect of GD on visceral adiposity and the underlying molecular mechanisms using zebrafish and mouse obesity models. GD ameliorated dyslipidemia and suppressed the accumulation of visceral adipose tissue (VAT) in adult obese zebrafish. Transcriptomic analysis by RNA sequencing of GD-treated adult zebrafish revealed that GD upregulated UCP1-related pathways. Further, we performed mouse experiments and found that GD intake (2 mg/g body weight/day) was associated with lowered plasma triglyceride and total cholesterol levels, decreased VAT accumulation, and improved adipocyte hypertrophy with the upregulation of *Ucp1* expression in white adipose tissue at both the mRNA and protein levels. Taken together, these results indicate that GD improves visceral adiposity by upregulating UCP1 expression, providing a novel perspective on combating obesity.

## Introduction

Obesity is becoming a significant global public health issue due to rapid increases in its prevalence and consequent health threats. According to the most recent fact sheet published by the World Health Organization, >1.9 billion adults were overweight, and of these, > 650 million were obese in 2016 (1). Data involving prevalence in the next generation showed that > 378 million children under the age of 19 were overweight or obese, which has risen > 4-fold from 1975 to 2016. Progressed or severe obesity increases the risk of comorbidities, including cardiovascular disease, type 2 diabetes, hypertension, dyslipidemia, and certain types of cancers (2, 3). Obesity is defined as the deposition of excessive body and ectopic fat. Excess energy is mainly stored in white adipose tissue (WAT) as triglycerides (4). Another type of adipose tissue, brown adipose tissue (BAT), functions to dissipate energy as heat through uncoupling protein-1 (UCP1) (5–7). WAT can transform into the BAT phenotype in response to appropriate stimuli, which are primarily mediated by high levels of UCP1 (8). This phenomenon is termed the “browning process” and results in beige- or brown-like adipose tissue (9, 10). Studies have demonstrated that browning of WAT with increased UCP1 expression exerts anti-obesity effects in rodent models (11). Thus, UCP1 is the best-characterized marker of the WAT browning process and is a good indicator to evaluate possible anti-obesity effects of chemicals or natural products.

Zebrafish (*Danio rerio*) is a well-established animal model for human diseases (12, 13). In 2010, we developed a zebrafish obesity model that mimics the pathology of human obesity by overfeeding (14). This model exhibits increased plasma triglyceride (TG) levels and hepatic steatosis combined with dysregulated lipid metabolism pathways, demonstrating that zebrafish is a suitable model for studying human obesity. Using this model, numerous active natural products and functional foods with anti-obesity effects were identified (15–21).

The health benefits of food-derived bioactive oligopeptides have been reported, including anti-inflammation (22, 23), anti-fatigue (24), gastroprotective effects (25), and hypoglycemic and hypolipidemic effects (26). Of these, globin digest (GD), a bioactive oligopeptide derived from porcine hemoglobin by acidic protease treatment (27), has been approved as a functional food in China and Japan. Previous studies have revealed that GD exerts a hypoglycemic effect in mice (28) and improves postprandial hyperlipidemia in rodents and dogs (27). A subsequent clinical trial involving healthy individuals also demonstrated suppressed postprandial serum triglyceride and chylomicron levels after GD intake (29). Recently, we performed a small-scale anti-obesity screening for natural products using zebrafish obesogenic test (30). Among the hit natural products, GD was identified to have lipid-lowering effects. However, a systemic evaluation of the effect of GD on visceral adiposity and the elucidation of the underlying molecular mechanisms have yet to be carried out. Here, we orally administered GD to adult zebrafish and performed RNA-sequencing (RNA-Seq) of hepatic tissues to investigate the underlying molecular mechanisms. We further tested GD in a mouse model of obesity to validate these results.

## Materials and Methods

### Ethics Statement

All animal procedures were approved by the Ethics Committee of Mie University, Tsu, Japan. Animal experiments were performed following the Japanese Animal Welfare Regulatory Practice Act on Welfare and Management of Animals (Ministry of Environment of Japan) and complied with international guidelines.

### Animals and Husbandry

Zebrafish (AB strain) were purchased from the Zebrafish International Research Center (ZIRC, OR, USA) and maintained at our facility under standard laboratory conditions (31). The zebrafish were fed GEMMA Micro 75, 150, and 300 (Skretting, Fontaine-les-Vervins, France) according to their developmental stages or body length. Wild-type Institute of Cancer Research (ICR) mice were purchased from Japan SLC, Inc. (Hamamatsu, Japan) and housed at the Institute of Laboratory Animals at Mie University (permission number: 28-4).

### Globin Digest

GD, with a total protein content of > 91 and <8% free amino acids, was purchased from MG Pharma Inc. (Osaka, Japan). GD is an oligopeptide mixture containing > 10 kinds of 3–5 amino acid residues, and the molecular weights of the peptides range between 100 and 1,500 u. GD is a white, odorless powder that is soluble in water.

### GD Administration to Adult Zebrafish

For oral administration of GD to adult zebrafish, 10% GD-containing zebrafish food was prepared using gluten as a carrier material, as previously described (32). The overfeeding experiment was designed and performed as previously reported (20, 30). Briefly, 3-month-old female zebrafish were randomly assigned to three groups with five fish per 2 L tank: (1) normal feeding group (NF) was fed a normal diet (gluten granules; 2 mg/fish/day) throughout the total of 3 weeks of the experiment and fed with 5 mg cysts/fish/day of *Artemia* during the third week; (2) overfeeding group (OF) was fed a normal diet (2 mg/fish/day) for 2 weeks, followed by 1 week of continuous normal diet and overfeeding with 60 mg cysts/fish/day of *Artemia*; (3) GD group was fed GD-containing gluten granules (2 mg/fish/day) at a dose of 250 μg/g body weight/day for 3 weeks and overfed with 60 mg cysts/fish/day of *Artemia* during the third week. The feeding details are shown in Supplementary Table 1. Normal and GD-containing diets were fed to zebrafish 30 min before *Artemia* feeding. The water circulation system was stopped for 2 h during feeding, and leftover foodstuff was removed by vacuuming to avoid water pollution. Body weights were measured once a week. At the end of the experiment, zebrafish were anesthetized, and blood was collected to measure fasting blood glucose, plasma TG, and plasma total cholesterol (TCHO) (33, 34). Subsequently, the fish were euthanized in an ice-water bath, and 3D-micro-computed tomography (CT) scans were performed using an *in vivo* System R_mCT 3D-micro-CT scanner (Rigaku, Tokyo, Japan). 3D images were reconstructed using i-View type R software (J. Morita Mfg, Kyoto, Japan) and analyzed using CT Atlas Metabolic Analysis ver. 2.03 software (Rigaku).

### RNA Isolation, RNA-Sequencing, and Bioinformatic Analyses

Fish were subjected to laparotomy, and hepatic tissues were collected by surgical manipulation. Total RNA was extracted and purified using TRIzol reagent (Life Technologies, Carlsbad, CA, USA) and QIAGEN RNeasy Mini-prep Kit (Qiagen, Hilden, Germany) (35). DNase digestion was performed on the column membranes to eliminate DNA contamination. The concentration of total RNA was measured using an Eppendorf spectrophotometer (BioPhotometer, Eppendorf, Hamburg, Germany). Ribosome RNA deletion and library construction were performed as previously described (20). The resulting libraries were sequenced using an Ion PGM system (Life Technologies). Signal processing, base calling, and adapter sequence trimming were performed using Torrent Suite software v.4.0.1.

Bioinformatic analysis was performed using CLC Genomics Workbench software 11.0.1 (Qiagen). The UCSC genome was used to map sequencing reads to the *Danio rerio* annotated genome, build GRCz10. Gene expression values were detected and normalized using the transcripts per million (TPM) algorithm. Expression data were exported from CLC Genomics Workbench as Microsoft Excel spreadsheets. We converted all non-human genes to human orthologs according to the Ensembl gene ortholog database (http://www.ensembl.org/biomart/martview). An enrichment false discovery rate (FDR) *q*-value <0.05 was considered statistically significant. After statistical tests, gene set enrichment analysis (GSEA) and sub-network enrichment analysis (SNEA) was performed using Pathway Studio 9.0 (Elsevier, Amsterdam, Netherlands).

### 3T3-L1 Adipocyte Differentiation Assay

Mouse 3T3-L1 preadipocytes (DS Pharma Biomedical, Osaka, Japan) were seeded in a 96-well plate in Dulbecco's modified Eagle medium-high glucose medium (Gibco, Gaithersburg, MD, USA) supplemented with 10% fetal bovine serum (FBS; Sigma Chemical Company, St. Louis, MO, USA) and antibiotics (100 U/mL penicillin and 100 μg/mL streptomycin) and incubated at 37°C under 5% CO_2_. Two days post-confluence, the cells were stimulated to differentiate into adipocytes by transferring them into adipocyte differentiation medium (ADM; DS Pharma Biomedical) for 6 days. The cells were maintained in ADM for an additional 2 days with or without 10 mg/mL GD. After GD treatment, the intracellular lipid content was measured using the AdipoRed Assay Reagent (Lonza, Walkersville, MD, USA) according to the manufacturer's instructions. Fluorescence images were taken using the BZ-X710 fluorescence microscope (Keyence, Tokyo, Japan). Intracellular lipid accumulation was quantified using the Victor2 multilabel plate reader (Ex 485 nm/Em 590 nm; PerkinElmer, Boston, MA, USA). After the AdipoRed assay, a cell viability assay was performed using the CellTiter-Glo Luminescent Cell Viability Assay (Promega, Madison, WI, USA) according to the manufacturer's instructions. For gene expression analysis, cells were seeded in 6-well plates, differentiated, treated with 10 mg/mL GD as described above, and collected for subsequent total RNA extraction.

### Mouse Experiments

Six-week-old male ICR mice were randomly assigned to four groups of ten mice each: (1) normal diet (ND) group was fed the CLEA Rodent Diet CE-7 (CLEA Japan, Tokyo, Japan) supplemented with 2.5 % gluten (*w/w*); (2) ND + GD group was fed a normal diet supplemented with 2.5% GD; (3) High fat diet (HFD) group was fed a HFD (60% of energy from fat; Test Diet 58Y1; TestDiet, Richmond, IN, USA) supplemented with 2.5% gluten; (4) HFD + GD group was fed a HFD supplemented with 2.5% GD. The experimental duration was 2 weeks. Body weight, fasting blood glucose, and food intake was measured weekly. At the end of the experiment, mice were euthanized by over-anesthesia with isoflurane (Pfizer, Pearl River, NY, USA). 3D micro-CT scans were performed as described above, and then liver, epididymal white adipose tissue (eWAT), and smooth muscle tissues were dissected for subsequent histology and qPCR analysis.

### Quantitative Reverse Transcription PCR

Total RNA from the liver, visceral adipose, and skeletal muscle tissues from zebrafish and mouse, or 3T3-L1 cells was extracted and purified using TRIzol reagent and QIAGEN RNeasy Mini-prep Kit, as described above. cDNA was synthesized from 500 ng total RNA using the ReverTra Ace qPCR RT Kit (Toyobo, Osaka, Japan). Quantitative reverse transcription PCR (RT-qPCR) was performed using Power SYBR Green Master Mix (Applied Biosystems, Foster City, CA, USA) and the ABI Stepone Plus Real-Time PCR System (Applied Biosystems, Foster City, CA, USA), according to the manufacturer's instructions. Relative mRNA levels were determined using 18S ribosomal RNA (*18s*) as an endogenous control gene. Sequences of the primers used for PCR amplification are listed in Supplementary Table 2.

### Fluorescent Immunohistochemical Staining

Mouse eWAT was collected and fixed using 4% formaldehyde solution in PBS (PFA; Histo-Fresh; Falma, Tokyo, Japan) at 4°C for 24 h. Fixed eWAT was then embedded in paraffin and cut into 3-μm sections. FIHC was performed according to the manufacturer's protocol (https://www.abcam.com/tag/ihc%20protocols). In brief, microtome sections were rehydrated and subjected to antigen retrieval using sodium citrate buffer in a presser cooker. The sections were incubated with primary antibody against mouse *Ucp1* (1:500; Abcam, ab234430, Cambridge, UK) overnight at 4°C. After rinsing with Tris-buffered saline with 0.025% Triton X-100 (TBST), the sections were incubated with a fluorescence-conjugated secondary antibody [anti-rabbit IgG (H + L), F(ab')2 Fragment, Alexa Fluor® 488 Conjugate; Cell Signaling Technology, Beverly, MA, USA] at room temperature (25°C) for 1 h and mounted with ProLong Gold Antifade Mountant with DAPI (Thermo Fisher Scientific, Waltham, MA, USA). Images were captured using a BZ-X710 fluorescence microscope (GFPfilter). Areas of adipocyte cells were quantified using ImageJ software (Fiji distribution, version 1.52p, National Institute of Health, Bethesda, MD, United States).

### Statistical Analysis

All data were analyzed using Student's *t*-test or one-way analysis of variance (ANOVA) with the Bonferroni–Dunn multiple comparison procedure, depending on the number of comparisons, using GraphPad Prism version 9 (GraphPad Software, San Diego, CA, USA). Results with *p* < 0.05 were considered statistically significant.

## Results

### GD Suppressed Visceral Adipose Tissue Accumulation in Zebrafish

**Figure 1:**
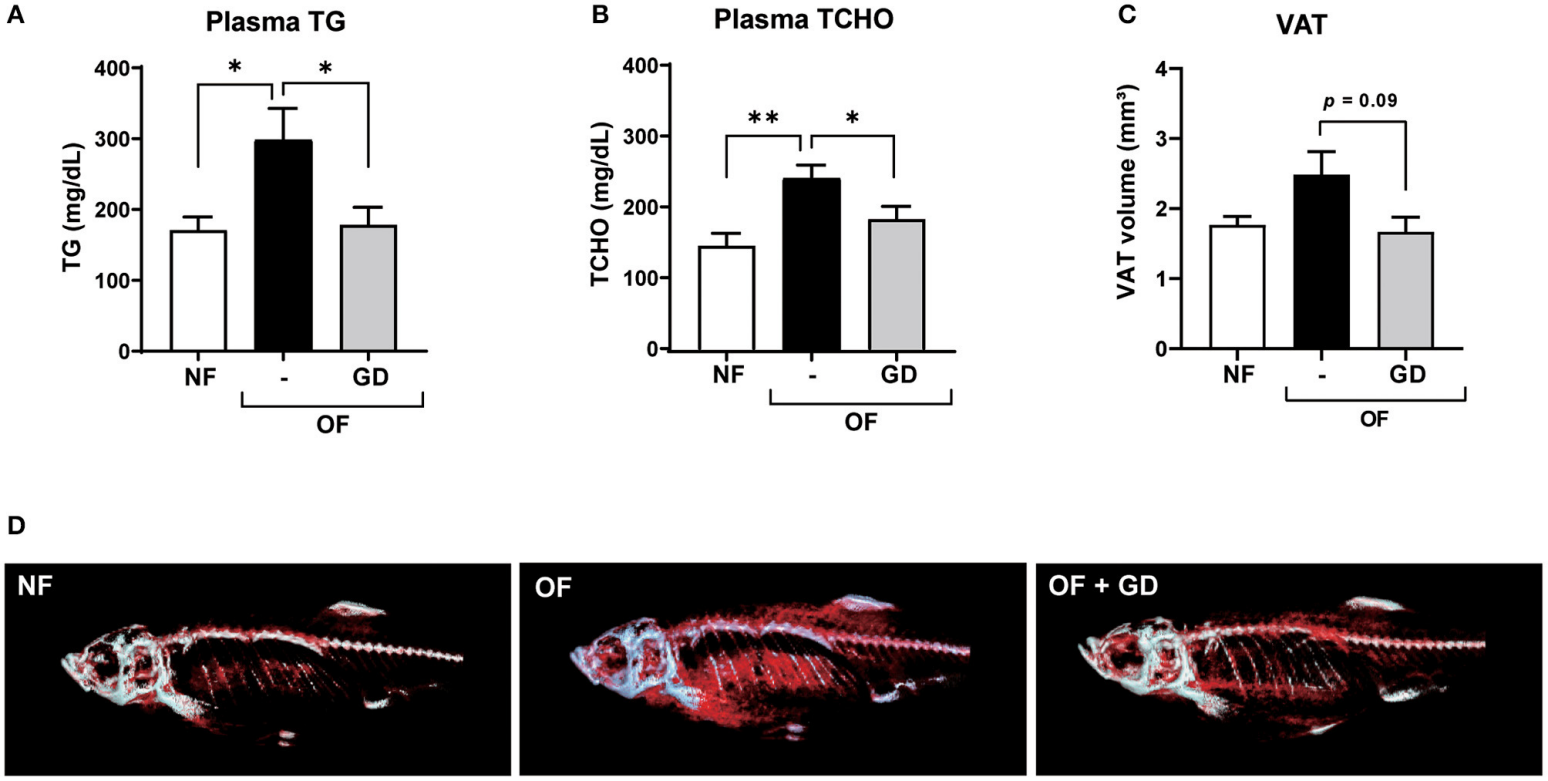
Effects of GD pre-administration in diet-induced obese adult zebrafish. GD suppressed an increase in plasma triglyceride (TG) **(A)** and plasma total cholesterol (TCHO) **(B)** levels in OF zebrafish. **(C)** GD reduced the visceral adipose tissue (VAT) volume in OF zebrafish. **(D)** Representative images to show VAT (red) after 3D micro-CT analysis. Gray color indicates skeletal bone. *p < 0.05, **p < 0.01 vs. OF, *n* = 5, error bars indicate SD.

The results of the zebrafish obesogenic test showed that GD significantly decreased the VAT volume in the experimental group (−29%; *p* < 0.05) compared with that of the juvenile control zebrafish (Supplementary Figure 1). We then orally administered GD (250 μg/g body weight/day) to adult zebrafish. Zebrafish in the GD group were fed with a normal diet supplemented with 10% GD-containing food for 2 weeks, followed by 1 week of overfeeding and a continuous GD-containing diet. This short-term overfeeding significantly increased the body weights of the OF and GD group animals, compared to those of the NF (*p* < 0.05; Supplementary Figures 2A,B). There were no statistically significant differences in changes in body weight and fasting blood glucose (FBG) levels between the OF and GD-treated groups (Supplementary Figure 2). However, GD administration significantly suppressed plasma TG levels (298 ± 44 mg/dL in OF vs. 178 ± 25 mg/dL in the GD group; *p* < 0.05; Figure 1A) and TCHO abundance (242 ± 18 mg/dL in OF vs. 183 ± 18 mg/dL in the GD group; *p* < 0.05; Figure 1B). In addition, the 3D-micro-CT analysis revealed a decrease in VAT volume in the GD group compared to the OF group (2.5 ± 0.3 mm^3^ in OF vs. 1.7 ± 0.2 mm^3^ in the GD group; *p* = 0.09; Figure 1C). Typical 2D images of zebrafish in the three groups constructed by CT analysis are shown in Figure 1D. Corresponding to the improvement in visceral adiposity, GD reduced adipose tissue accumulation more than in the OF group.

### GD Upregulated *ucp1* Expression in Adult Zebrafish Liver

**Figure 2:**
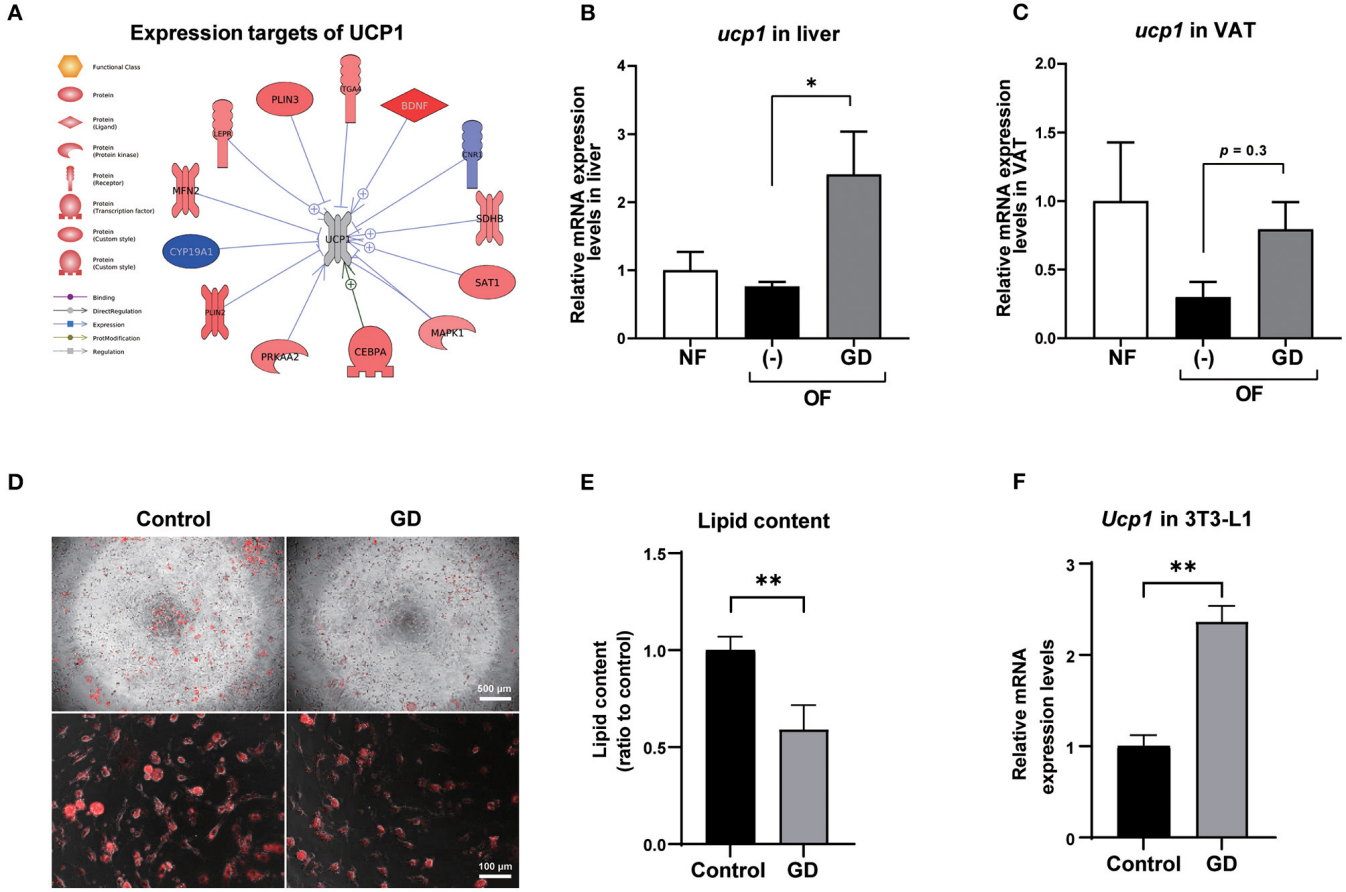
Expression levels of UCP1 in zebrafish liver and VAT. **(A)** Protein expression targets of UCP1 were identified by Pathway Studio comparing altered genes in GD-treated zebrafish with those of OF zebrafish. The red and blue colors denote genes with increased and decreased expression, respectively. The gray color of UCP1 indicates UCP1 was not detected in RNA-Seq analysis. Gene expression level changes of *ucp1* in zebrafish liver **(B)** and VAT **(C)** were validated by qPCR analysis. *p < 0.05 vs. OF, *n* = 5, error bars indicate SD. **(D)** Representative images of AdipoRed staining of differentiated control adipocytes and GD-treated adipocytes. 3T3-L1 cells were differentiated and treated with 10 mg/mL GD for 2 days and processed for AdipoRed staining. Mature adipocytes were photographed at the magnification of 40 × (upper panels) and 200 × (lower panels). The red color indicates lipid accumulation. **(E)** Effect of 10 mg/mL GD on intracellular lipid accumulation in 3T3-L1 adipocytes. Cellular lipid content was measured by AdipoRed assay. **p < 0.01 vs. control. **(F)** GD-induced mRNA expression level change of *Ucp1* in 3T3-L1 cells. **p < 0.01 vs. control, *n* = 6, error bars indicate SD.

To investigate the molecular mechanism underlying the anti-adiposity effects of GD, we performed RNA-Seq transcriptomic analysis using adult obese zebrafish liver tissues with or without GD treatment. We identified altered pathways and gene networks using GSEA (36), followed by SNEA (37). The top 20 cell process-related genetic pathways enriched by GD-regulated genes revealed that GD intake significantly activated digestion and gastrointestinal absorption-related pathways, including eating behavior, gastric emptying, intestinal motility, gastrointestinal motility, and pancreatic juice secretion (*p* < 1E-04; Table 1). Eighty-five such pathways are listed in Supplementary Table 3. SNEA extracts a gene-gene expression regulation network from global literature to determine sub-networks for protein expression targets, which are displayed as a central hub (seed) with a group of genes (gene sets) that share common biological functions. The top 15 protein expression targets (*p* < 0.05) regulated by GD compared with OF zebrafish are listed in Table 2. UCP1, a mitochondrial protein mainly expressed in the inner mitochondrial membrane of brown adipocytes, was among these predicted functional gene set seeds (38). As a central seed, the relationships of UCP1 with its 13 neighbors (expression targets, binding partners, and protein modification targets) are shown in Figure 2A. Red color indicates upregulated, and blue color indicates down-regulated expression in GD-treated zebrafish compared to OF zebrafish. Since the gray color of UCP1 suggested no detection in the RNA-Seq assay (weak expression or cut off by the *p*-value during bioinformatics analysis), we performed a qPCR analysis to confirm the expression levels of *ucp1* using cDNA samples synthesized from the total RNAs used in RNA-Seq analysis. The mRNA expression level of *ucp1* showed a significant increase (3.2-fold, *p* < 0.05) in GD-treated zebrafish liver compared with that in OF zebrafish liver (Figure 2B), whereas there was no significant difference in the VAT tissues of the two groups (*p* = 0.3, Figure 2C).

**Table 1:** Top 20 cell process-related genetic pathways/groups enriched by GD-regulated genes (*p* < 1E-04).

**Gene set seed**	**Total # of Neighbors**	**Overlap**	***p*-value**
Eating behavior	336	99	2.22E-09
Estrous cycle	251	88	1.83E-08
Neuromodulation	104	41	3.38E-08
Gastric emptying	151	48	5.09E-08
Pressor response	245	85	6.25E-08
Intestine motility	183	58	6.25E-08
Natriuresis	160	52	7.15E-08
Gastrointestinal motility	152	55	7.33E-08
Excitability	737	228	7.54E-08
Membrane steady potential	185	63	1.05E-07
Potassium conductance	89	32	1.77E-07
Satiety	209	66	1.87E-07
Grooming behavior	121	38	1.98E-07
Diuresis	172	47	3.35E-07
Cardiovascular deconditioning	192	65	4.03E-07
Micturition	116	37	4.29E-07
REM sleep	149	49	4.78E-07
Pancreatic juice secretion	85	33	5.01E-07
Transmission of nerve impulse	845	269	5.89E-07
Vocalization	101	30	6.61E-07

**Table 2:** Top 15 protein expression targets regulated by GD in OF zebrafish compared with those of OF zebrafish.

**Gene set seed**	**Total # of Neighbors**	**Overlap**	**Measured neighbors**	***p*-value**
bHLH factor	82	6	VIP; BDNF; HES6; HEYL; NR4A2; MYF5	0.0007
HOXB1	45	5	HBE1; HOXA1; PKNOX1; RARA; RARG	0.0044
NRP1	93	6	SGK1; SLC31A1; PTPRC; EIF4E; GPNMB; NR4A2	0.0091
NR4A1	187	9	BDNF; HTR2A; CEBPA; HMGCR; MAPK9; HIF1A; MAPK1; NR4A2; RARG	0.0162
Polysome	121	14	CSRP3; CAMK2A; BDNF; ATF3; NPM1; EIF4E; AK6; EIF1AX; EIF4B; HNRNPD; ATF4; MTDH; HDLBP; ELAVL2	0.0184
ACTA2	768	35	ANGPTL5; BDNF; PLIN2; ATF3; HTR2A; SGK1; GADD45A; HMGCR; S1PR2; SOAT1; FOXF2; AOC3; RHOC; EIF4E; CDH5; CSTB; GSK3B; GNL3; TGM2; SLC2A1; PECAM1; HMGB1; MAPK9; ANXA2; MSRA; ARG2; HIF1A; PARP1; USP10; MAPK1; GATA6; FGL2; CNR1; KL; CDH1	0.0209
UCP1	363	13	BDNF; PLIN2; PLIN3; CEBPA; SAT1; MFN2; PRKAA2; ITGA4; LEPR; SDHB; MAPK1; CNR1; CYP19A1	0.0240
MAP2	121	5	BDNF; AACS; RARA; HMGB1; RARG	0.0249
PDGFRB	131	10	HTR2A; FOXF2; AOC3; LETM1; TGM2; PECAM1; HIF1A; ESR2; CYP19A1; DRD4	0.0282
CD44	411	16	PLOD2; SGK1; MTHFD2; HMGCR; S1PR2; GSK3B; GNL3; TGM2; SACM1L; NUS1; PLOD1; HMGB1; PDHA1; HIF1A; MAPK1; CDH1	0.0298
UCP3	89	7	MYOD1; NFIL3; CS; NR4A1; PPARD; NPY; MYF5	0.0319
TUBB3	152	7	MRC1; BDNF; GSK3B; HIF1A; MAPK1; CDH1; CYP19A1	0.0324
GAP43	157	10	APOD; NEFL; BDNF; ATF3; MAPK12; GSK3B; ANXA2; MAPK1; SOCS3; CYP19A1	0.0327
FOXP1	52	5	RUNX1; KRAS; NR4A1; MSX2; HOXC9	0.0345

### GD Upregulated *Ucp1* Expression in 3T3-L1 Adipocytes and Mice eWAT Tissue

To confirm whether *Ucp1* upregulation due to GD administration is common in mammals, we performed an *in vitro* experiment to determine the change in *Ucp1* expression levels in 3T3-L1 cells, a mouse preadipocyte cell line. Six days after the start of adipocyte differentiation, GD (10 mg/mL) was administered to differentiated 3T3-L1 cells for 2 days, followed by AdipoRed staining. GD administration significantly suppressed lipid accumulation in 3T3-L1 cells (Figures 2D,E). The lipid content in GD-treated cells was decreased by 0.6-fold compared to the untreated differentiated adipocyte control (*p* < 0.01, Figure 2E), and there was no significant effect on cell viability after GD treatment. The qPCR analysis revealed a marked increase in *Ucp1* mRNA expression induced by 10 mg/mL of GD compared to the control (2.4-fold, *p* < 0.01, Figure 2F).

**Figure 3:**
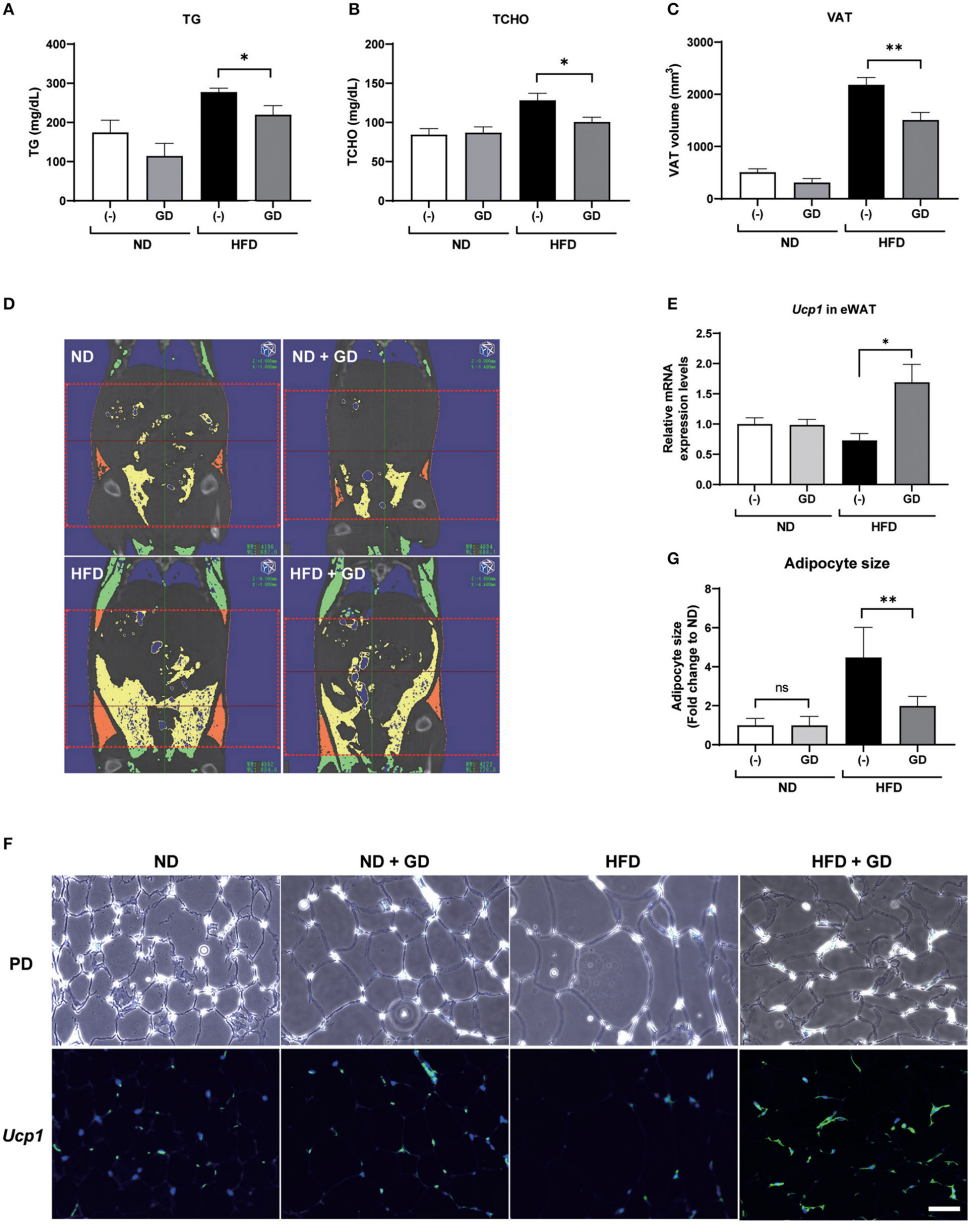
Effects of GD intake on ICR mice on a high-fat diet (HFD). Change in plasma TG **(A)**, plasma TCHO **(B)**, and VAT volume **(C)** after 2 weeks of GD administration. **(D)** Represents micro-CT images of the four groups. The red dotted lines demarcate the area for which VAT volume was measured (from the xiphoid process of the sternum to the hip joint). Yellow indicates the visceral adipose tissue, and orange indicates the subcutaneous adipose tissue. **(E)** GD-induced *Ucp1* expression in mice eWAT. **(F)** Fluorescent immunohistochemical staining (FIHC) with anti-mouse *Ucp1* antibody in eWAT sections. PD, phrase difference images; Scale bar = 50 μm. **(G)** Average adipocyte size (*n* = 30) of the four mouse groups in **(F)**. *p < 0.05, **p < 0.01 vs. HFD, *n* = 10, error bars indicate SD.

We further orally administered GD (2.5% *w/w* in the HFD; ~2 mg/g body weight/day) to wild-type ICR mice. Short-term feeding with HFD for 2 weeks significantly increased the body weights of HFD mice compared to those of the ND and ND + GD groups (*p* < 0.05), while there was no significant difference in body weight changes and FBG levels between HFD and HFD with GD-administered groups (Supplementary Figures 3A,B). The food intake was not different between HFD and HFD + GD groups, which indicated that GD did not suppress appetite (Supplementary Figure 3C). To verify the effects of GD on lipid metabolism in the blood, we measured plasma TG and TCHO levels. GD treatment resulted in reduced plasma TG levels compared to the HFD group (*p* < 0.05, Figure 3A). The GD group also showed significantly reduced TCHO levels compared to the HFD group (*p* < 0.05, Figure 3B). Three-dimensional micro-CT analysis revealed that the VAT volume in the HFD group was significantly higher (*p* < 0.001) than that in the ND group, and GD treatment significantly reduced the VAT volume compared with the HFD group (*p* < 0.01, Figure 3C). Representative micro-CT images of the four groups are shown in Figure 3D. To determine whether GD supplementation, as observed in zebrafish, elevated the *Ucp1* expression levels in mice, we performed qPCR analysis using mouse liver and eWAT with or without GD administration. Signals corresponding to *Ucp1* in liver tissues were not detected due to low tissue specificity in the mouse liver (data not shown). Nevertheless, a significant increase in the *Ucp1* expression levels in the eWAT tissue of the GD-treated group was observed compared to that in the HFD group (0.73- and 1.69-fold vs. ND group, respectively; *p* < 0.05; Figure 3E).

We further performed FIHC using an anti-*Ucp1* antibody to investigate the protein expression levels in eWAT of mice induced by GD (Figure 3F). Phase difference (PD) images are shown in the upper lane. Increased adipocyte size (hypertrophy) was markedly induced by HFD compared with that in ND groups (*p* < 0.001) but was significantly decreased by GD treatment (*p* < 0.001, Figure 3G). The lower lane of Figure 3F shows fluorescent images of the four groups. As expected, GD-treated mice displayed upregulated *Ucp1* levels in eWAT. In addition, we found that the *Ucp1* signals were also induced in BAT tissues of GD-treated mice (Supplementary Figure 4).

## Discussion

As the zebrafish model is a powerful tool for anti-obesity drug discovery, we used juvenile zebrafish for test material screening and adult diet-induced obese zebrafish to assess the hit materials. Using this evaluation system, we detected GD as an anti-obesity material by using small-scale drug screening (30). In the present study, we further investigated the effects of GD on the suppression of visceral adiposity and the possible molecular mechanism involved. Conclusions derived from the experiments with the zebrafish were eventually confirmed by the observations in the obese mouse model, providing a new target for combating human obesity. Based on these results, we have decided to plan a human trial to test the potential function of GD in improving human adiposity. Ten years ago, Bowman and Zon proposed a novel phenotype-driven drug discovery pipeline (39). They suggested using zebrafish first to perform high-throughput screening and to identify the lead compound in any given issue, and then optimizing the lead compound by mammalian modeling, and finally, by using human/clinical trials. This strategy leads to a fast and cost-effective route for drug development. Our current study conforms to this proposal and provides an example for expediting anti-obesity drug development.

Genome-wide gene expression analysis using transcriptome sequencing provides a convenient tool to detect molecular mechanisms underlying the anti-obesity effects of GD. Table 1 shows that GD intake significantly regulated pathways related to digestion and gastrointestinal absorption including gastric emptying, intestinal motility, and gastrointestinal motility. This result was consistent with the conclusion of a previous study that administered ^14^C-labeled lipids to mice and found that the radioactivity level in feces excreted from GD-administered mice was twice that of the control mice (27). Among the top 15 protein expression targets regulated by GD in obese zebrafish liver tissue (Table 2), several gene set seeds have been reported to be involved in energy and lipid metabolism. Platelet-derived growth factor receptor β (PDGFRB) is an adipocyte progenitor marker, and PDGFR signaling determines adipocyte progenitor commitment to white adipogenesis (40); nuclear receptor subfamily 4, group A, member 1 (NR4A1), and CD44, both of which have been demonstrated as negative regulators of UCP1 (41, 42), were also modulated by GD. These results revealed the possible pathway mechanisms of GD that contribute to the anti-obesity effects that were previously observed. In addition to these gene set seeds, we noticed the measured neighbors of UCP1 regulated by GD in Figure 2A. GD administration upregulated the mRNA expression of brain-derived neurotrophic factor (BDNF), spermidine/spermine N1-acetyltransferase (SAT1), mitofusin 2 (MFN2), and protein kinase AMP-activated catalytic subunit alpha 2 (PRKAA2). BDNF is an immediate and direct modulator of energy expenditure (by inducing UCP1) and glucose metabolism in obese diabetic animals (43). SAT1 is the key enzyme involved in the catabolism of polyamines. SAT1 transgenic mice with activated polyamine catabolism showed reduced WAT mass, a high basal metabolic rate, and improved glucose tolerance (44). MFN2 is a GTPase enzyme that controls mitochondrial dynamics and is a mediator of mitochondria to lipid droplet interactions, influencing lipolytic processes and whole-body energy homeostasis (45). PRKAA2 is a subunit of AMP-activated protein kinase (AMPK), which is known to be a cellular energy sensor (46). One study reported that hepatic AMPK activation blocks white adipose tissue expansion in diet-induced obese mice (47). Considering these results in zebrafish and referring to the available literature, we hypothesized that GD improves visceral adiposity by upregulating UCP1.

To confirm this hypothesis, we administered GD in mice. The short-term administration of GD showed a trend to decrease the VAT volume, and the adipocyte hypertrophy was significantly ameliorated (Figure 3). The mRNA expression and protein levels of *Ucp1* in eWAT were also upregulated by GD administration. Studies have demonstrated that the browning process accompanied by increased *Ucp1* expression exerts anti-obesity effects in rodent models^11^. Our findings indicate that GD administration upregulates UCP1, which may be associated with an active browning process that finally results in anti-obesity effects. A recent report revealed that silk peptide prevents HFD-induced obesity and induces WAT browning by activating AMPK and increasing UCP1 expression in mice (48). These similar findings regarding GD function provide further evidence that dietary bioactive peptides may act as a preventive or therapeutic agent for obesity treatment.

Besides UCP1, another member of the thermogenesis family, UCP3, is also regulated by GD administration (Table 2). UCP3 is mainly expressed in the skeletal muscles of mammals and is known to substantially contribute to lipid metabolism and whole-body energy metabolism (49, 50). Choi et al. demonstrated that the overexpression of UCP3 in skeletal muscle protected mice from HFD-induced defects, including increased whole-body fat mass, hepatic steatosis, and insulin resistance, through an increase in the whole-body energy expenditure (51). In addition, UCP3 can protect mitochondria against fat oxidation by increasing fatty acid delivery (52). In our study, GD-mediated expression of UCP3 in the zebrafish liver may be linked to increased fatty acid oxidation and energy expenditure. Another member of the UCP family, UCP2, is reported to be upregulated in the mouse skeletal muscle by GD feeding, which may be related to its beneficial effects on glucose metabolism (28). In this study, significantly increased *Ucp2* mRNA expression was also observed in the skeletal muscle tissue of GD-treated mice (Supplementary Figure 5). In addition to the role of UCP2 as a glucose transporter in skeletal muscle, Ucp2 is also involved in the regulation of energy metabolism and obesity (53). A positive correlation between weight loss and UCP2 expression has been reported (54). Although no weight loss was found in the GD-treated zebrafish and obese mice models, some myogenesis biomarkers were found to be upregulated in the skeletal muscle of mice (data not shown). This finding may explain why GD inhibits VAT accumulation without weight loss. We are currently working on evaluating the effects of GD on promoting muscle growth and hope to publish our findings soon. Taken together, we hypothesize that GD might systemically activate UCPs (UCP1, UCP2, and UCP3) to increase energy expenditure in the body, in addition to VAT and muscles; however, further studies are needed.

In the present study, we demonstrated that GD reduced VAT accumulation and ameliorated hyperlipidemia in obese zebrafish and mouse models. Transcriptomic analysis using zebrafish revealed that UCP1 is a novel target of GD. Furthermore, mRNA expression and protein levels of *Ucp1* in the eWAT of obese mice were also upregulated in response to GD administration. Our study results provide evidence that GD supplementation inhibits visceral adiposity through UCP1 upregulation, suggesting its novel role in anti-adiposity therapy.

## Data Availability Statement

The datasets presented in this study can be found in online repositories. The names of the repository/repositories and accession number(s) can be found below: NCBI SRA repository, accession: PRJNA691414, https://www.ncbi.nlm.nih.gov/sra/PRJNA691414.

## Ethics Statement

The animal study was reviewed and approved by Ethics Committee of Mie University.

## Author Contributions

LZ, YSh, HN, and IM performed the experiments and analyzed the data. LZ and JK performed bioinformatic analysis. LZ prepared the original draft, and YSh reviewed and edited the manuscript. YK, D-CC, RT, and YSa provided the GD and advised the present study. YSh, LJ, and NN conceived and designed the experiments. All authors have read and approved the final manuscript.

## Conflict of Interest

YK, D-CC, and LJ were employees of Rohto Pharmaceutical Co., Ltd., a pharmaceutical company. RT and YSa are employees of MG Pharma Inc., a pharmaceutical company (a member of the Rohto Pharmaceutical group). The remaining authors declare that the research was conducted in the absence of any commercial or financial relationships that could be construed as a potential conflict of interest.

## Publisher's Note

All claims expressed in this article are solely those of the authors and do not necessarily represent those of their affiliated organizations, or those of the publisher, the editors and the reviewers. Any product that may be evaluated in this article, or claim that may be made by its manufacturer, is not guaranteed or endorsed by the publisher.

## References

[1] WHO. Obesity and Overweight. (2020). Available online at: https://www.who.int/en/news-room/fact-sheets/detail/obesity-and-overweight (accessed June 11, 2020).

[2] Pi-Sunyer X. The medical risks of obesity. Postgrad Med. (2009) 121:21–33. 10.3810/pgm.2009.11.2074 19940414 PMC2879283

[3] Piche ME Tchernof A Despres JP. Obesity phenotypes, diabetes, and cardiovascular diseases. Circ Res. (2020) 126:1477–500. 10.1161/CIRCRESAHA.120.316101 32437302

[4] Weisberg SP Mccann D Desai M Rosenbaum M Leibel RL Ferrante AW. Obesity is associated with macrophage accumulation in adipose tissue. J Clin Investig. (2003) 112:1796–808. 10.1172/JCI200319246 14679176 PMC296995

[5] Frontini A Cinti S. Distribution and development of brown adipocytes in the murine and human adipose organ. Cell Metab. (2010) 11:253–6. 10.1016/j.cmet.2010.03.004 20374956

[6] Chechi K Nedergaard J Richard D. Brown adipose tissue as an anti-obesity tissue in humans. Obes Rev. (2014) 15:92–106. 10.1111/obr.12116 24165204

[7] Marlatt KL Ravussin E. Brown adipose tissue: an update on recent findings. Curr Obes Rep. (2017) 6:389–96. 10.1007/s13679-017-0283-6 29101739 PMC5777285

[8] Lo KA Sun L. Turning WAT into BAT: a review on regulators controlling the browning of white adipocytes. Biosci Rep. (2013) 33:e00065. 10.1042/BSR20130046 23895241 PMC3764508

[9] Cao L Choi EY Liu X Martin A Wang C Xu X. White to brown fat phenotypic switch induced by genetic and environmental activation of a hypothalamic-adipocyte axis. Cell Metab. (2011) 14:324–38. 10.1016/j.cmet.2011.06.020 21907139 PMC3172615

[10] Shabalina IG Petrovic N De Jong JM Kalinovich AV Cannon B Nedergaard J. UCP1 in brite/beige adipose tissue mitochondria is functionally thermogenic. Cell Rep. (2013) 5:1196–203. 10.1016/j.celrep.2013.10.044 24290753

[11] Wu J Cohen P Spiegelman BM. Adaptive thermogenesis in adipocytes: is beige the new brown? Genes Dev. (2013) 27:234–50. 10.1101/gad.211649.112 23388824 PMC3576510

[12] Dooley K Zon LI. Zebrafish: a model system for the study of human disease. Curr Opin Genet Dev. (2000) 10:252–6. 10.1016/S0959-437X(00)00074-5 10826982

[13] Zang LQ Maddison LA Chen WB. Zebra fish as a model for obesity and diabetes. Front Cell Dev Biol. (2018) 6:e00091. 10.3389/fcell.2018.00091 30177968 PMC6110173

[14] Oka T Nishimura Y Zang L Hirano M Shimada Y Wang Z. Diet-induced obesity in zebrafish shares common pathophysiological pathways with mammalian obesity. BMC Physiol. (2010) 10:21. 10.1186/1472-6793-10-21 20961460 PMC2972245

[15] Tainaka T Shimada Y Kuroyanagi J Zang L Oka T Nishimura Y. Transcriptome analysis of anti-fatty liver action by Campari tomato using a zebrafish diet-induced obesity model. Nutr Metab (Lond). (2011) 8:88. 10.1186/1743-7075-8-88 22152339 PMC3275548

[16] Hiramitsu M Shimada Y Kuroyanagi J Inoue T Katagiri T Zang LQ. Eriocitrin ameliorates diet-induced hepatic steatosis with activation of mitochondrial biogenesis. Sci Rep. (2014) 4:3708. 10.1038/srep03708 24424211 PMC3892443

[17] Zang L Shimada Y Kawajiri J Tanaka T Nishimura N. Effects of Yuzu (Citrus junos Siebold ex Tanaka) peel on the diet-induced obesity in a zebrafish model. J Funct Foods. (2014) 10:499–510. 10.1016/j.jff.2014.08.002

[18] Zang LQ Shimada Y Tanaka T Nishimura N. Rhamnan sulphate from Monostroma nitidum attenuates hepatic steatosis by suppressing lipogenesis in a diet-induced obesity zebrafish model. J Funct Foods. (2015) 17:364–70. 10.1016/j.jff.2015.05.041

[19] Nakayama H Shimada Y Zang LQ Terasawa M Nishiura K Matsuda K. Novel anti-obesity properties of palmaria mollis in zebrafish and mouse models. Nutrients. (2018) 10:1401. 10.3390/nu10101401 30279329 PMC6213011

[20] Zang LQ Shimada Y Nakayama H Kim Y Chu DC Juneja LR. RNA-seq based transcriptome analysis of the anti-obesity effect of green tea extract using zebrafish obesity models. Molecules. (2019) 24:3256. 10.3390/molecules24183256 31500159 PMC6767142

[21] Matsuura N Zang LQ Nishimura N Shimada Y. Lacto-fermented cauliflower fungus (*Sparassis crispa*) ameliorates hepatic steatosis by activating beta-oxidation in diet-induced obese zebrafish. J Med Food. (2020) 23:803–10. 10.1089/jmf.2019.4571 32466711

[22] Huang F Wang J Yu F Tang Y Ding G Yang Z. Protective effect of meretrix meretrix oligopeptides on high-fat-diet-induced non-alcoholic fatty liver disease in mice. Mar Drugs. (2018) 16:39. 10.3390/md16020039 29360762 PMC5852467

[23] Xu M Chen Q Fan R Wang J Li Y. Anti-inflammation effect of small molecule oligopeptides prepared from Panax ginseng C. A Meyer in rats. Molecules. (2019) 24:858. 10.3390/molecules24050858 30823424 PMC6429476

[24] Liu R Wu L Du Q Ren JW Chen QH Li D. Small molecule oligopeptides isolated from walnut (*Juglans regia* L.) and their anti-fatigue effects in mice. Molecules. (2018) 24:45. 10.3390/molecules24010045 30583565 PMC6337178

[25] Liu R Hao YT Zhu N Liu XR Kang JW Mao RX. The gastroprotective effect of small molecule oligopeptides isolated from walnut (*Juglans regia* L.) against ethanol-induced gastric mucosal injury in rats. Nutrients. (2020) 12:1138. 10.3390/nu12041138 32325708 PMC7231309

[26] Wei Y Zhang R Fang L Qin X Cai M Gu R. Hypoglycemic effects and biochemical mechanisms of Pea oligopeptide on high-fat diet and streptozotocin-induced diabetic mice. J Food Biochem. (2019) 43:e13055. 10.1111/jfbc.13055 31591749

[27] Kagawa K Matsutaka H Fukuhama C Watanabe Y Fujino H. Globin digest, acidic protease hydrolysate, inhibits dietary hypertriglyceridemia and Val-Val-Tyr-Pro, one of its constituents, possesses most superior effect. Life Sci. (1996) 58:1745–55. 10.1016/0024-3205(96)00156-7 8637399

[28] Nakaoka F Sasakawa Y Yamamoto K Nakao M Nakamura M Tong C. Anti-diabetic effects of globin digest and its active ingredient Leu-Ser-Glu-Leu in ICR mice, streptozotocin-induced diabetic mice and KK-Ay mice. Life Sci. (2010) 86:424–34. 10.1016/j.lfs.2010.01.014 20109472

[29] Kagawa K Matsutaka H Fukuhama C Fujino H Okuda H. Suppressive effect of globin digest on postprandial hyperlipidemia in male volunteers. J Nutr. (1998) 128:56–60. 10.1093/jn/128.1.56 9430602

[30] Nakayama H Hata K Matsuoka I Zang L Kim Y Chu D. Anti-obesity natural products tested in juvenile zebrafish obesogenic tests and mouse 3T3-L1 adipogenesis assays. Molecules. (2020) 25:5840. 10.3390/molecules25245840 33322023 PMC7764013

[31] Westerfield M. The Zebrafish Book, A Guide for the Laboratory Use of Zebrafish (Danio rerio). 5th ed. Eugene: University of Oregon Press (2007).

[32] Zang L Morikane D Shimada Y Tanaka T Nishimura N. A novel protocol for the oral administration of test chemicals to adult zebrafish. Zebrafish. (2011) 8:203–10. 10.1089/zeb.2011.0726 22181663

[33] Zang LQ Shimada Y Nishimura Y Tanaka T Nishimura N. A novel, reliable method for repeated blood collection from aquarium fish. Zebrafish. (2013) 10:425–32. 10.1089/zeb.2012.0862 23668933

[34] Zang LQ Shimada Y Nishimura Y Tanaka T Nishimura N. Repeated blood collection for blood tests in adult zebrafish. Jove J Visual Exp. (2015) 102:e53272. 10.3791/53272 26383512 PMC4692578

[35] Peterson SM Freeman JL. RNA isolation from embryonic zebrafish and cDNA synthesis for gene expression analysis. J Vis Exp. (2009) 7:1470. 10.3791/1470 19684565 PMC3152201

[36] Subramanian A Tamayo P Mootha VK Mukherjee S Ebert BL Gillette MA. Gene set enrichment analysis: a knowledge-based approach for interpreting genome-wide expression profiles. Proc Natl Acad Sci USA. (2005) 102:15545–50. 10.1073/pnas.0506580102 16199517 PMC1239896

[37] Kotelnikova E Yuryev A Mazo I Daraselia N. Computational approaches for drug repositioning and combination therapy design. J Bioinform Comput Biol. (2010) 8:593–606. 10.1142/S0219720010004732 20556864

[38] Chang SH Song NJ Choi JH Yun UJ Park KW. Mechanisms underlying UCP1 dependent and independent adipocyte thermogenesis. Obes Rev. (2019) 20:241–51. 10.1111/obr.12796 30450758

[39] Bowman TV Zon LI. Swimming into the future of drug discovery: *in vivo* chemical screens in zebrafish. ACS Chem Biol. (2010) 5:159–61. 10.1021/cb100029t 20166761 PMC4712380

[40] Gao ZG Daquinag AC Su F Snyder B Kolonin MG. PDGFR alpha/PDGFR beta signaling balance modulates progenitor cell differentiation into white and beige adipocytes. Development. (2018) 145:dev15561. 10.1242/dev.155861 29158445 PMC6514402

[41] Kanzleiter T Schneider T Walter I Bolze F Eickhorst C Heldmaier G. Evidence for Nr4a1 as a cold-induced effector of brown fat thermogenesis. Physiol Genomics. (2005) 24:37–44. 10.1152/physiolgenomics.00204.2005 16219868

[42] Liu LF Kodama K Wei K Tolentino LL Choi O Engleman EG. The receptor CD44 is associated with systemic insulin resistance and proinflammatory macrophages in human adipose tissue. Diabetologia. (2015) 58:1579–86. 10.1007/s00125-015-3603-y 25952479

[43] Tsuchida A Nonomura T Ono-Kishino M Nakagawa T Taiji M Noguchi H. Acute effects of brain-derived neurotrophic factor on energy expenditure in obese diabetic mice. Int J Obes. (2001) 25:1286–93. 10.1038/sj.ijo.0801678 11571589

[44] Pirinen E Kuulasmaa T Pietila M Heikkinen S Tusa M Itkonen P. Enhanced polyamine catabolism alters homeostatic control of white adipose tissue mass, energy expenditure, and glucose metabolism. Mol Cell Biol. (2007) 27:4953–67. 10.1128/MCB.02034-06 17485446 PMC1951486

[45] Boutant M Kulkarni SS Joffraud M Ratajczak J Valera-Alberni M Combe R. Mfn2 is critical for brown adipose tissue thermogenic function. EMBO J. (2017) 36:1543–58. 10.15252/embj.201694914 28348166 PMC5452040

[46] Garcia D Shaw RJ. AMPK: mechanisms of cellular energy sensing and restoration of metabolic balance. Mol Cell. (2017) 66:789–800. 10.1016/j.molcel.2017.05.032 28622524 PMC5553560

[47] Garcia D Hellberg K Chaix A Wallace M Herzig S Badur MG. Genetic liver-specific AMPK activation protects against diet-induced obesity and NAFLD. Cell Rep. (2019) 26:192–208.e196. 10.1016/j.celrep.2018.12.036 30605676 PMC6344045

[48] Lee K Jin H Chei S Lee JY Oh HJ Lee BY. Dietary silk peptide prevents high-fat diet-induced obesity and promotes adipose browning by activating AMP-activated protein kinase in mice. Nutrients. (2020) 12:201. 10.3390/nu12010201 31941008 PMC7019986

[49] Vidal-Puig A Solanes G Grujic D Flier JS Lowell BB. UCP3: an uncoupling protein homologue expressed preferentially and abundantly in skeletal muscle and brown adipose tissue. Biochem Biophys Res Commun. (1997) 235:79–82. 10.1006/bbrc.1997.6740 9196039

[50] Nabben M Hoeks J. Mitochondrial uncoupling protein 3 and its role in cardiac- and skeletal muscle metabolism. Physiol Behav. (2008) 94:259–69. 10.1016/j.physbeh.2007.11.039 18191161

[51] Choi CS Fillmore JJ Kim JK Liu ZX Kim S Collier EF. Overexpression of uncoupling protein 3 in skeletal muscle protects against fat-induced insulin resistance. J Clin Invest. (2007) 117:1995–2003. 10.1172/JCI13579 17571165 PMC1888566

[52] Schrauwen P Saris WH Hesselink MK. An alternative function for human uncoupling protein 3: protection of mitochondria against accumulation of nonesterified fatty acids inside the mitochondrial matrix. FASEB J. (2001) 15:2497–502. 10.1096/fj.01-0400hyp 11689475

[53] Schrauwen P Hesselink M. UCP2 and UCP3 in muscle controlling body metabolism. J Exp Biol. (2002) 205:2275–85. 10.1242/jeb.205.15.2275 12110661

[54] Cortes-Oliveira C Nicoletti C De Souza Pinhel M De Oliveira B Quinhoneiro D Noronha N. UCP2 expression is associated with weight loss after hypocaloric diet intervention. Eur J Clin Nutr. (2017) 71:402–6. 10.1038/ejcn.2016.185 27759071

