# Supplementary material for: Globin Digest Improves Visceral Adiposity Through UCP1 Upregulation in Diet-Induced Obese Zebrafish and Mice
Source: Front Nutr. 2021 Sep 27;8:650975. doi: 10.3389/fnut.2021.650975 (PMC8502801; doi:10.3389/fnut.2021.650975)
Supplement: Supplementary file 1 [file Data_Sheet_1.pdf]

Supplementary Material

1 Supplementary Methods

1.1 Zebrafish Obesogenic Test (ZOT) using Juvenile Zebrafish

ZOT was performed as previously reported, with minor modifications (Tingaud-Sequeira et al., 2011;Zang et al., 2019). In brief, juvenile fish with a standard length of approximately 1 cm (3–4 weeks old) were selected and placed into a 6-well plate containing 5 mL 0.3 × Danieau's solution (17.4 mM NaCl, 0.21 mM KCl, 0.12 mM $MgSO_4$, 0.18 mM $Ca(NO_3)_2$, and 1.5 mM 4-(2-hydroxyethyl)-1-piperazinyl-ethane-2-sulfonic acid (HEPES); pH 7.6). Fish were fed 0.1% boiled chicken egg yolk on the first day of ZOT. On the second day, the egg yolk solution was replaced with fresh 0.3 × Danieau's solution, and the juvenile fish were exposed to 10 μg/mL GD for one day without feeding. The control group without GD exposure was also starved. The volume of visceral adipose tissue was measured before and after GD treatment by Nile Red staining, as previously described (Zang et al., 2019). Fluorescence images were obtained using a BZ-X710 fluorescence microscope (Keyence Japan, Osaka, Japan). Quantification of Nile red positive signals was performed using ImageJ software (Fiji distribution, v.1.52p, National Institutes of Health, Bethesda, MD, USA).

Reference

Tingaud-Sequeira, A., Ouadah, N., and Babin, P.J. (2011). Zebrafish obesogenic test: a tool for screening molecules that target adiposity. *J Lipid Res* 52**,** 1765-1772.

Zang, L.Q., Shimada, Y., Nakayama, H., Kim, Y., Chu, D.C., Juneja, L.R., Kuroyanagi, J., and Nishimura, N. (2019). RNA-seq Based Transcriptome Analysis of the Anti-Obesity Effect of Green Tea Extract Using Zebrafish Obesity Models. *Molecules* 24.

2 Supplementary Figures and Tables

2.1 Supplementary Figures

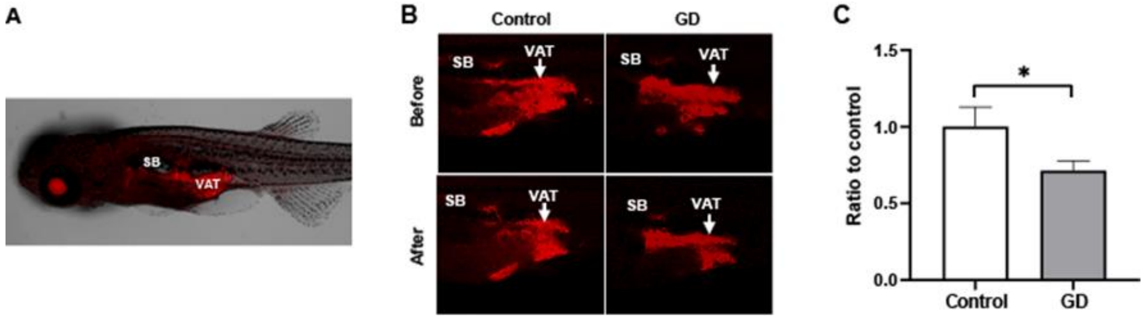

Supplementary Figure S1. Globin digest (GD) suppressed visceral adipose tissue (VAT) accumulation in zebrafish juveniles. (A) Lateral view of a juvenile zebrafish under a fluorescence

microscope after Nile Red staining. Nile Red-positive areas indicate VAT, and SB indicates the swimming bladder. (B) Representative VAT images from live juvenile zebrafish selected in the zebrafish obesogenic test after Nile Red staining. Images were taken before and after 24 h exposure with or without GD. (C) Quantification of the intensity of Nile Red positive area of control and GD groups. The Y-axis indicates the ratio of Nile Red staining before and after GD administration for 24 h. * $p < 0.05$ vs. control, n = 5, error bars indicate SD.

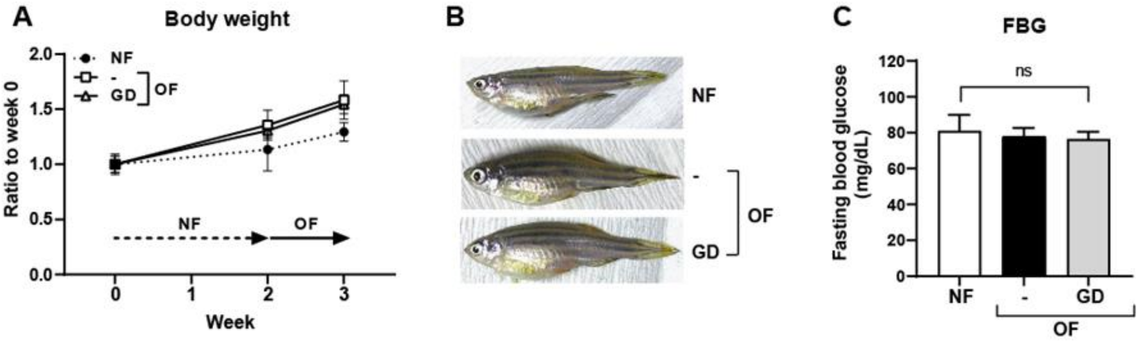

Supplementary Figure S2. Effects of GD pre-administration in adult zebrafish with diet-induced obesity. (A) Body weight change during the 3-week experiment. NF, normal feeding; OF, overfeeding. (B) Representative images of zebrafish that were subjected to NF, OF, and OF with GD. (C) Fasting blood glucose (FBG) levels in the three groups.

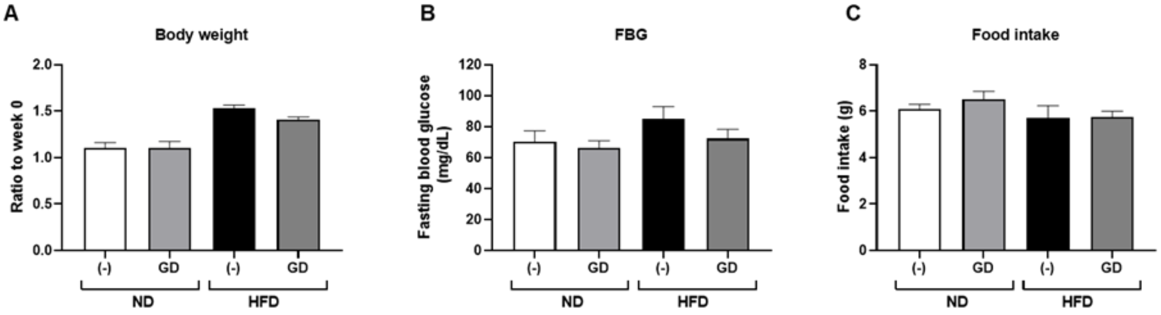

Supplementary Figure S3. Effects of GD intake in ICR mice on a high-fat diet (HFD). The graphs represent changes in body weight (A), FBG (B), and food intake (C) after the mice were fed ND and HFD with or without GD supplementation. n = 10, error bars indicate SD.

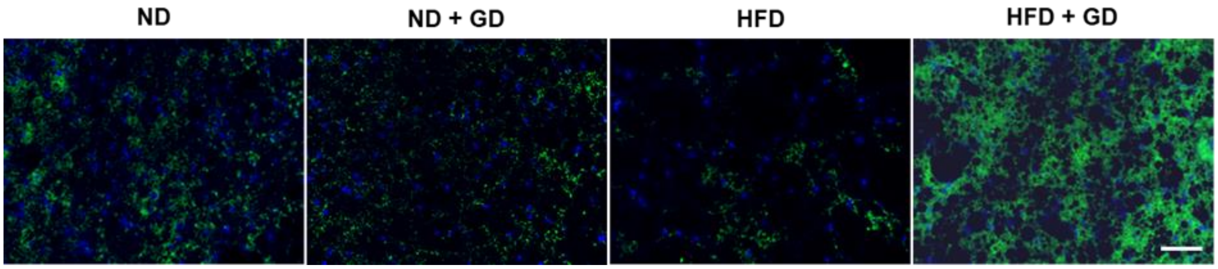

Supplementary Figure S4. Fluorescent immunohistochemical staining (FIHC) with anti-mouse *Ucp1* antibody in mice BAT sections.

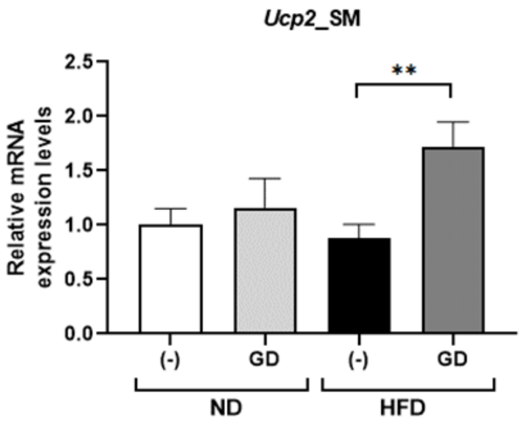

Supplementary Figure S5. GD-induced mRNA expression level change of *Ucp2* in mice skeletal muscle tissues. ** $p < 0.01$, n = 5, error bars indicate SD.

2.2 Supplementary Tables

Supplementary Table S1. Feeding details for the zebrafish study.

	The first two weeks			The third week						
Diet	Gluten granules (mg/fish/day)	GD-containing Gluten granules (mg/fish/day)	Total calories	*Artemia* (mg cysts/fish/day)	Gluten granules (mg/fish/day)	GD-containing Gluten granules (mg/fish/day)	Total calories	Protein (g)	Carbohydrate (g)	Fat (g)
NF	2	-	2.2	5	2	-	22.2	0.20	0.10	0.06
OF	2	-	2.2	60	2	-	152.2	1.52	0.77	0.43
OF + GD	-	2	2.8	60	-	2	152.8	1.52	0.77	0.43

Supplementary Table S2. Primer sequences for qPCR.

	Symbol	Accession number	Forward primer (5' - 3')	Reverse primer (5' - 3')	Product size (bp)
**Zebrafish**	*ucp1*	NM_199523	GGTGTGGGCAGACGATACAA	CTCGTGATGTTCGGCAGAGT	85
	18s	NM_001098396	GCCTGCGGCTTAATTTGACT	ACCACCCACAGAATCGAGAAA	97
**Mouse**	*Ucp1*	NM_009463	CCTGCCTCTCTCGGAAACAA	GTAGCGGGGTTTGATCCCAT	115
	Ucp2	NM_011671	CAAGGGGTTCATGCCTTCCT	AAAGGTGCCTCCCGAGATTG	117
	18S	NR_003278	GGCCGTTCTTAGTTGGTGGAGCG	CTGAACGCCACTTGTCCCTC	133

Supplementary Table S3. Cell process-related genetic pathways/groups enriched by GD-regulated genes (p < 1E-04).

Gene Set Seed	Total # of Neighbors	Overlap	Measured Neighbors	p-value
eating behavior	336	99	CAMK2A;VIP;ADORA2A;BDNF;NTRK2;FGF5;CRH;FABP7;CCKAR;HRH3;OXTR;HRH2;SLC5A11;FAS;PRKCQ;NKX2-1;OPRM1;EDN1;SST;HTR2B;PRKAR2B;SIM1;NMUR2;UNC119;APOA4;FHL1;HTR2A;ESR1;GCK;PTPN18;ACACA;PKNOX1;NAMPT;TNFRSF1A;PRKAA2;MYC;CNR2;GSK3B;NUCB2;SRD5A2;NR4A3;ACHE;COPA;LEPR;ANGPTL3;GRN;PRLR;TRPA1;CPT1A;ATP1A1;PARP1;CA6;ESR2;C3;NR1D1;RNMT;CCR2;ITLN1;EIF2S1;IGF2;RPS6KB1;STAT3;NR1H4;MTOR;PRCP;PTPN1;HTR6;PPARA;IRS2;TRPV1;GRIN2B;SOCS3;CNR1;IRS1;SPX;ETV5;NELL2;SLC24A4;NOS2;HTR1A;MC4R;TPH1;SHOX2;QRFPR;GPR119;SLC6A3;AVPR1A;ESRRG;OPRK1;GRPR;DRD2;INS;NPFFR1;NPY;GPER1;CCKBR;MC3R;DRD4;BSX1	2.22E-09
estrous cycle	251	88	CAMK2A;VIP;REN;PGF;HOXA10;BDNF;WNT5A;CRH;OXTR;FAS;OPRM1;TLR2;EDN1;EGF;PAQR7;SST;CRABP1;EDNRB;CD109;NTRK1;CFTR;ESR1;AKR1B1;NAMPT;PTGES3;SLC2A1;ACHE;LEPR;HSD17B12;GSN;ANXA2;PLA2G4A;SRD5A1;FLT1;NIP7;FST;PTGER2;ATP1A1;ASAH1;NT5E;ESR2;INHBA;KDR;CBR1;NR1D1;THOP1;AQP1;CDK4;ANXA1;TGFB1;HSD17B1;GRM1;GJA1;CTGF;VEGFA;RBP4;CYR61;IGF1R;NCAM1;CREBRF;PROKR2;SCARB1;NELL2;GDF9;PPARD;NOS2;MC4R;CNGA2;RBP1;CHRNA7;AQP3;FSHR;SLC6A3;AVPR1A;FGF7;LPL;DLG4;OPRK1;DBH;GPR173;GJB4;SYT9;WNT4;INS;NPY;NTSR1;GPER1;CYP19A1	1.83E-08
neuromodulation	104	41	VIP;KCNJ10;ADORA2A;BDNF;CRH;HRH3;HRH2;OPRM1;EDN1;VIPR1;SST;TACR1;SLC6A2;GPX1;HTR2A;SLC25A27;CNR2;ACHE;CXCR4;NT5E;MAPK1;SLC30A7;SIGMAR1;APP;TGFB1;VIPR2;GRM1;CNR1;TRHR;HTR1A;IL10;NPPC;CHRNA7;CASR;SLC6A3;AVPR1A;OPRK1;DRD2;INS;NPY;GPER1	3.38E-08
gastric emptying	151	48	VIP;CRH;CCKAR;OXTR;HRH2;OPRM1;EGF;PAQR7;SST;TACR1;TAS1R3;APOA4;HTR2A;SERPINB1;DPP4;PDE5A;FOXF2;CNR2;NUCB2;MYLK;ACHE;COPA;LEPR;TRPA1;CHRM3;AMY2A;KDR;TPH2;DGAT1;GNA11;ADRA2A;TRPV1;F13A1;CNR1;NOS2;TRHR;MC4R;IL10;NPPC;GPR119;CASR;SCTR;GRPR;DRD2;INS;ADRB1;NPY;CCKBR	5.09E-08
pressor response	245	85	VIP;REN;PTGER1;ADORA2A;BDNF;NTRK2;ENPEP;CRH;CCKAR;HRH3;HRH2;OPRM1;EDN1;EGF;SST;ADRA1B;HTR2B;EDNRB;CYP1B1;TACR1;SIM1;GRIN1;SLC6A2;NCF1;HTR2A;ESR1;AGTRAP;RNPEP;PDE5A;PTPRC;CYBA;CNR2;RHOA;CBS;ACHE;MYH11;NAA35;CHRM3;FLT1;PTGER2;RNLS;ADRA1D;AGTR2;ESR2;MAPK1;DAO;MAPK3;SAR1A;THOP1;GNA11;TGFB1;CCR2;STAT3;ATP5J;PTPN1;TBXAS1;CYBB;PTEN;TRPV1;ADRA2B;ADRA1A;IGF1R;TMEM133;CNR1;ANPEP;PRKCB;NOS2;TRHR;HTR1A;MC4R;IL10;NPPC;PNMT;CHRNA7;AVPR1A;SLC35F3;OPRK1;C3AR1;DRD2;INS;ADRB1;NPY;MC3R;CCKBR;PTH1R	6.25E-08
intestine motility	183	58	VIP;FFAR2;ADORA2A;BDNF;CALCR;NTRK2;CRH;CCKAR;HRH3;HRH2;OPRM1;EDN1;EGF;FFAR3;SST;ENO2;TACR1;TAS1R3;APOA4;FABP6;CFTR;FHL1;KRAS;ACTG2;CNR2;MYLK;ACHE;COPA;MYH11;TRPA1;CHRM3;MAPK1;TPH2;F2R;RAN;TRPC6;F2RL1;ARRB2;STAT3;KIT;BMP7;LRRK2;TRPV1;CNR1;NOS2;ANO1;HTR1A;NPPC;TPH1;GPR119;CASR;SLC6A3;OPRK1;GRPR;DRD2;INS;NPY;NTSR1	6.25E-08
natriuresis	160	52	VIP;REN;KCNJ10;PTGER1;ADORA2A;ENPEP;CRH;OXTR;HRH2;EDN1;EGF;CORIN;SLC5A2;EDNRB;TACR1;SLC4A8;AGTRAP;SGK1;DPP4;PDE5A;TNFRSF1A;GPR182;PPP5C;PRLR;PTGER2;AGTR2;MAPK1;TGFB1;MTOR;VEGFA;NPR3;FURIN;TRPV1;SLC8A1;OCLN;NFAT5;ANPEP;KL;PPARD;NOS2;SLC8A2;NPPC;CASR;AVPR1A;TGFB2;DRD2;INS;ADRB1;NPY;MC3R;CCKBR;DRD4	7.15E-08
gastrointestinal motility	152	55	VIP;ADORA2A;BDNF;CRH;CCKAR;HRH2;CNN1;OPRM1;TLR2;EDN1;EGF;SST;ADORA2B;EDNRB;BMP2;TACR1;HTR2A;SERPINB1;DPP4;SCG2;HSPE1;CNR2;RHOA;HSPA9;CBS;MYLK;ACHE;COPA;LEPR;TRPA1;CHRM3;PTGER2;KDR;F2R;ADRA2A;F2RL1;GJA1;KIT;TRPV1;SLC8A1;CNR1;SPX;SCN5A;NOS2;ANO1;HTR1A;MC4R;NMUR1;NPPC;OPRK1;DRD2;INS;NPY;NTSR1;CCKBR	7.33E-08
excitability	737	228	CSRP3;CAMK2A;VIP;KCNJ10;PTGER1;SCN4B;EN2;ADORA2A;BDNF;WNT5A;SCN1B;ERC2;KCNT2;KCNH1;NTRK2;PSMB8;TRPM4;CRH;FXYD6;CCKAR;HRH2;KCNK13;KCNK18;SLC5A11;PRKCQ;CAV3;OPRM1;KCNC4;KCNN3;EDN1;P2RY12;CALHM1;KCNMB2;SST;ENO2;GNAQ;GLRA3;KCNB2;ADRA1B;KCNJ11;HTR2B;ADORA2B;NLGN4X;TMEM38A;TACR1;GRIN2C;JPH3;KCNQ3;CACNA1S;GRIA3;GRIN1;KCNG4;UNC13A;NTRK1;TMC2;CFTR;HTR2A;ESR1;SGK1;ALOX12;PDE5A;S1PR2;TNFRSF1A;PVALB;ABCC9;EIF2AK2;EIF4E;CNR2;SYN2;RHOA;GSK3B;HSPB1;NUCB2;SIL1;KIF5B;CBS;SACM1L;KCNJ13;CACNA1E;ACHE;ADIPOR2;HMGB1;LEPR;RAP1A;RORA;TRPA1;KCNK15;CXCR4;NIPA2;CLIC1;SYP;VPS52;PRKCD;SRD5A1;CHRM3;ATF4;VAPB;TESC;IKBKB;PARP1;NT5E;AGTR2;ESR2;CACNA1C;MAPK1;SIGMAR1;SPTBN1;NEU1;PIK3CB;KDR;TPH2;KCNJ18;CALR;SP4;F2R;MAPK3;SATB1;DTNBP1;PDXP;GPHN;NCEH1;GNA11;RNMT;CLCN3;APP;EPS15;ADRA2A;TOP1;TRPC6;TGFB1;F2RL1;CCR2;GRM2;DARS;ARRB2;GALNS;GRM1;UBE3A;MEF2A;IGF2;GJA1;USP9X;NOTCH1;MTOR;HTR6;VEGFA;HOMER1;CHRNA3;TCF7L2;SP3;ATP2B1;NFATC3;PTEN;NFIC;IRS2;IL1R1;SEMA4D;RASD1;ERBB4;EHMT2;HTR5A;RBFOX1;FURIN;TRPV1;BEST1;SYNGAP1;PRKN;PRKCE;SLC8A1;ADRA1A;GRIN2B;GRIK2;KCTD7;IGF1R;NCAM1;THBS4;TMEM266;SOCS3;CNR1;RGS7;LPAR2;LGI1;SLC1A2;SCN5A;CACNA1D;DPP6;ANO1;SCN8A;KCND1;TRHR;NAV1;NCAM2;KCNIP2;HTR1A;ADORA3;MC4R;GABRD;IL10;NPPC;TPH1;CHRNA7;TRPC1;CASR;SLC6A3;KCNC1;SLC6A1;ETV1;GRM6;NCS1;HTR1F;OPRK1;FGF14;NLGN2;ARHGEF9;KCNJ6;KCNK16;TGFB2;ERG;DRD2;INS;DCT;ADRB1;NPY;NTSR1;GPER1;CCKBR;CYP19A1;DRD4	7.54E-08

membrane steady potential	185	63	VIP;REN;KCNJ10;BDNF;KCNH1;TRPM4;CRH;HRH3;KCNK18;KCNC4;KCNN3;EDN1;SST;KCNB2;KCNJ11;TMEM38A;GRIN2C;KCNQ3;CFTR;HTR2A;SGK1;PVALB;ABCC9;CTH;MYLK;TRPA1;CHRM3;ATP1A1;CACNA1C;MAPK1;KCNJ18;PRMT1;MAPK3;APP;TRPC6;TGFB1;VIPR2;GRM1;UBE3A;GJA1;PICK1;TRPV1;KCTD7;CNR1;GLUL;CACNA1D;DPP6;NOS2;ANO1;SCN8A;HTR1A;KCNJ15;NPPC;KCNC1;SLC6A1;TRPV6;KCNJ6;ERG;DRD2;INS;ADRB1;NPY;DRD4	1.05E-07
potassium conductance	89	32	KCNJ10;CRH;CCKAR;OXTR;HRH2;KCNK18;FAS;OPRM1;EDN1;EGF;SST;GNAQ;KCNJ11;TACR1;KCNQ3;HTR2A;SGK1;PVALB;CHRM3;APP;GRM2;ARRB2;GRM1;KCTD7;CNR1;HTR1A;OPRK1;DRD2;INS;ADRB1;NPY;DRD4	1.77E-07
satiety	209	66	VIP;FFAR2;BDNF;NTRK2;CRH;CCKAR;HRH3;OXTR;SLC15A1;FAS;ISL1;OGN;OPRM1;FFAR3;SST;SIM1;GRIN2C;APOA4;HTR2A;ESR1;LGALS1;DPP4;ACACA;NAMPT;CNR2;NUCB2;DBI;COPA;LEPR;ANGPTL3;CPE;TRPA1;CPT1A;MAPK1;TPH2;ADRA2A;TGFB1;MARK4;STAT3;MTOR;PTPN1;HTR6;PPARA;IRS2;HTR5A;TRPV1;UCP3;CNR1;SPX;GCGR;HTR1A;MC4R;WNT10B;TSHR;GPR119;CASR;ESRRG;OPRK1;GRPR;DRD2;INS;NPY;GPER1;MC3R;CCKBR;DRD4	1.87E-07
grooming behavior	121	38	ADORA2A;BDNF;CRH;OXTR;OPRM1;EDN1;SST;HTR2B;TACR1;SIM1;HTR2A;ESR1;DAB1;DBN1;SYN2;GSK3B;GRN;MAPK1;MAPK3;TNFSF13B;GRM2;GRM1;VDR;CNR1;ADCY5;SLC1A2;NOS2;HTR1A;MC4R;IL10;SLC6A3;AVPR1A;OPRK1;DBH;GRPR;DRD2;INS;NPY	1.98E-07
diuresis	172	47	REN;KCNJ10;PTGER1;CRH;HRH2;OPRM1;EDN1;EGF;SST;CORIN;SLC5A2;EDNRB;TACR1;CFTR;CD63;SLC9A3R1;DPP4;PDE5A;TNFRSF1A;PTRH2;GPR182;AGTR2;ESR2;MAPK1;AQP1;TGFB1;NPR3;PPARA;FURIN;TRPV1;ADCY6;NFAT5;CNR1;NOS2;NPPC;CAPN2;AQP3;TSHR;CASR;AVPR1A;OPRK1;DRD2;INS;ADRB1;NPY;CCKBR;DRD4	3.35E-07
cardiovascular deconditioning	192	65	VIP;REN;ADORA2A;NTRK2;CRH;CCKAR;HRH2;ARRB1;OPRM1;GPR75;KCNN3;EDN1;SST;HTR2B;SLC5A2;ADORA2B;EDNRB;TACR1;SLC6A2;HTR2A;ESR1;DPP4;PDE5A;APOA1;PMVK;CTH;CNR2;PPP5C;ACHE;HMGB1;LEPR;MDK;TRPA1;NT5E;AGTR2;ESR2;MAPK1;LGALS3;APP;ADRA2A;TRPC6;F2RL1;ARRB2;MTOR;VEGFA;NPR3;PPARA;TRPV1;ADCY6;XDH;CNR1;HTR1A;MC4R;NPPC;SLC6A3;AVPR1A;OPRK1;DBH;INS;ADRB1;NPY;NTSR1;GPER1;MC3R;CCKBR	4.03E-07
micturition	116	37	VIP;REN;PTGER1;BDNF;CRH;HRH3;IL4I1;HRH2;EDN1;EGF;SST;SLC5A2;TACR1;SHH;NTRK1;ADA;DPP4;PDE5A;MB;TNFRSF1A;VIM;B2M;ACHE;SRD5A1;CHRM3;ADRA1D;AGTR2;INHBA;AQP1;GRM1;GJA1;ADRA1A;HTR1A;NPPC;DRD2;INS;NPY	4.29E-07
REM sleep	149	49	VIP;REN;ADORA2A;BDNF;NTRK2;CRH;HRH3;HRH2;OPRM1;TLR2;EGF;VIPR1;LHX6;SST;HTR2B;CFTR;ADA;HTR2A;NAMPT;TNFRSF1A;PVALB;CNR2;ACHE;CHRM3;CACNA1C;MAPK1;CSNK1E;F2R;MAPK3;APP;GRM2;HTR6;PER3;IL1R1;GRIN2B;NCAM1;CNR1;NOS2;HTR1A;IL10;SLC6A3;SLC6A1;NCS1;DBH;NLGN2;DRD2;INS;ADRB1;NPY	4.78E-07
pancreatic juice secretion	85	33	VIP;CRH;CCKAR;HRH2;ARRB1;OPRM1;EGF;SST;TACR1;TMPRSS15;CFTR;HTR2A;GCK;ODC1;DPP4;AQP8;DBI;PREP;COPA;CHRM3;PSMD4;F2RL1;NFE2L1;NPR3;CNR1;NPPC;GPR119;SCTR;GRPR;INS;NPY;NTSR1;CCKBR	5.01E-07
transmission of nerve impulse	845	269	CAMK2A;VIP;REN;KCNJ10;PTGER1;ADORA2A;BDNF;STMN1;WNT5A;SCN1B;CACNG4;KCNH1;NTRK2;CRH;FXYD6;CCKAR;HRH3;OXTR;HRH2;KCNK18;ESM1;OPRM1;KCNC4;KCNN3;EDN1;P2RY12;EGF;VIPR1;LHX6;CALHM1;CNTNAP1;KCNMB2;SST;GNAQ;KCNB2;CNTN2;HTR2B;PTN;TACR1;GRIN2C;CAMSAP2;CLDN19;KCNQ3;NFATC4;GRIA3;NPAS4;GRIN1;KIF5C;UNC13A;SHH;SLC4A8;CFTR;ADA;LYN;ATF3;HTR2A;ESR1;GCK;SGK1;DPP4;PDE5A;EHMT1;S1PR2;SLC7A10;DBN1;TNFRSF1A;RAB10;PVALB;EIF2AK2;EIF4E;CTH;SCG2;CNR2;RGS4;SYN2;RAB8A;RHOA;GSK3B;KIF5B;CBS;MYLK;KCNJ13;HLA-A;CACNA1E;ACHE;ADIPOR2;MYO5B;CNIH1;HMGB1;LEPR;CAMK1;RAP1A;PRLR;TRPA1;RAP1B;CXCR4;MSRA;PRKCD;ATF4;SMPD2;MARK2;CAPZA1;FSTL1;UGT8;CDC42;PARP1;ADRA1D;GPR89B;GOLGA2;AGTR2;GNB2;CA8;ESR2;CACNA1C;MAPK1;ACP1;SIGMAR1;SPTBN1;NEU1;NTN1;STK39;PIK3CB;PRMT1;DAO;JUN;F2R;HSD17B10;MAPK3;RALA;SATB1;DTNBP1;SEMA3C;GNA11;THBS1;CLCN3;APP;AQP1;SPTAN1;KIF13B;ADRA2A;TRPC6;TGFB1;F2RL1;CCR2;GRM2;ARRB2;GALNS;GRM1;VIPR2;FOXO3;UBE3A;PAFAH1B1;TFAM;TIAM1;TLN1;ATP5H;GJA1;STAT3;RPS6KB1;MTOR;PICK1;HTR6;VEGFA;NCDN;HOMER1;SV2A;TCF7L2;ADCY3;INPPL1;ATP2B1;CYBB;PPARA;PTEN;SIRT2;VTN;RGS12;IL1R1;RGS9;ERBB4;RBFOX1;FURIN;TRPV1;BEST1;VDAC1;PLCE1;PTPRZ1;SYNGAP1;PRKCE;PRICKLE1;SEMA3A;ZNRF1;GRIN2B;GRIK2;ATP1A3;VDR;PTK2;ENPP2;IGF1R;NCAM1;CHL1;NR4A1;KIF17;SOCS3;CNR1;RGS7;CRY1;SARM1;LLGL1;LGI1;GLUL;CDH10;SLC1A2;SCN5A;CACNA1D;KL;APBA2;DPP6;KCNC2;NOS2;GJB3;ANO1;SCN8A;CNTNAP4;GABBR1;KCND1;TRHR;BRSK1;SATB2;KCNIP2;HTR1A;ADORA3;MC4R;NMUR1;GABRD;IL10;NPPC;CHRNA7;PIWIL1;KCNH5;CASR;SLC6A3;KCNC1;SLC6A1;ETV1;AVPR1A;GRM6;EFNB3;GLDN;NCS1;HTR1F;DLG4;OPRK1;FGF14;DBH;NLGN2;KCNJ6;GRPR;DRD2;INS;MTNR1A;NPY;NTSR1;KCNIP4;GPER1;KCNAB1;BIN1;MTNR1B;LRFN1;DRD4	5.89E-07
vocalization	101	30	VIP;ADORA2A;BDNF;JAKMIP1;NTRK2;CRH;CCKAR;OXTR;OPRM1;SST;CDH6;NLGN4X;TACR1;HTR2A;ESR1;LRFN2;CACNA1C;GRM1;PTEN;CADM1;IGF1R;CNR1;HTR1A;CACNA2D3;AVPR1A;DLG4;OPRK1;DRD2;NTSR1;CYP19A1	6.61E-07
taste aversion	105	37	BDNF;NTRK2;CRH;HRH3;OPRM1;HTR2B;TACR1;TAS1R3;GRIN1;NTRK1;HTR2A;CEBPA;FOSL2;EIF2AK2;COPA;HMGB1;LEPR;TRPA1;EIF4EBP2;MAPK1;SIGMAR1;JUN;APP;GRM2;GRIN2B;CNR1;GABBR1;HTR1A;MC4R;SLC6A3;DLG4;OPRK1;GRPR;DRD2;INS;NPY;DRD4	8.44E-07

calcium mobilization	931	284	CAMK2A;VIP;PGF;DEF6;PTGER1;FFAR2;ADORA2A;LCK;BDNF;WNT5A;NTRK2;XCR1;PLSCR1;CD79A;CRH;CCKAR;HRH3;OXTR;IL4I1;HRH2;MYH7;ARRB1;FAS;PRKCQ;DMTN;OPRM1;SAA1;KCNN3;LTB4R;CASQ2;TLR2;EDN1;PIP4K2C;LCP2;P2RY12;EGF;GPR12;VIPR1;GPR84;SST;GNAQ;ADRA1B;KCNJ11;SPRY1;HTR2B;GATA1;CD180;ADORA2B;EDNRB;TACR1;P2RY13;NMUR2;DKK1;CD226;NTRK1;TNFRSF14;PDZK1;LGALS9;CFTR;ADA;SCIN;LYN;HTR2A;ESR1;GCK;LGALS1;PTPN6;HMGCR;SLC9A3R1;CORO1A;DPP4;ALOX12;HLA-B;S1PR2;PTPRC;AHCYL1;MFN2;TNFRSF1A;PVALB;PTRH2;HSPA5;UTS2R;CNR2;RGS4;HAX1;RHOA;GSK3B;TGM2;ZDHHC3;VAV3;MCAM;PECAM1;MYLK;NR4A3;SACM1L;TMED2;HSP90AA1;GRAP2;DBI;PDE3A;HMGB1;GRB2;LEPR;CAMK1;RAP1A;CKB;TRPA1;CXCR4;RAB27A;HIF1A;JAK3;PLCH1;RAC2;PRKCD;GPSM1;TRAP1;ZAP70;GNA13;GNA12;CHRM3;FLT1;SRI;SNAPIN;ITGA1;LGALS8;PTGER2;ITGAM;BTK;EPRS;CDC42;PPIB;EZR;PARP1;ADRA1D;PLPP1;PLCB4;SPRY4;AGTR2;DCN;ARHGDIB;BAX;RGS5;CCR9;MAPK1;PACSIN2;ACP1;DGKG;SIGMAR1;NEU1;PIK3CB;KDR;RAB5A;CALR;C3;CD81;FADD;F2R;PSMA3;PDLIM1;MAPK3;NQO1;PDIA4;PSEN2;GUSB;TRAF6;GNA11;INPP5D;RNMT;APP;PLEK;ADRA2A;ANXA1;TRPC6;KIF21A;SELENOT;TGFB1;F2RL1;CCR2;PTPN11;GRM2;SNW1;ARRB2;BSG;TMEM147;ST13;LAMC1;GRM1;ITGB2;IGF2;PTPRJ;ZDHHC7;GJA1;CTGF;ITPKB;STAT3;TFPI;MTOR;PGGT1B;PTPN1;PDIA3;VEGFA;KIT;RASA1;CFLAR;HOMER1;SEPN1;TLR5;ADCY3;NPR3;PIN1;ATP2B1;PTEN;SH3KBP1;AHNAK;LRRK2;NRIP1;RGS9;TRPV1;LRP1;PDE4D;PLCE1;FN1;PRKCE;SLC8A1;ADRA1A;VDR;F7;IGF1R;GM2A;NCAM1;XDH;PROKR2;CNR1;SLC24A3;IRS1;RGS7;SCARB1;LPAR2;CHRNB2;GCGR;SLC1A2;PRKCB;NPHS1;PPARD;NOS2;ARR3;TRHR;PTK6;RGS11;HTR1A;ADORA3;NMUR1;IL10;NPPC;TPH1;CHRNA7;TRPC1;TSHR;GPR119;CASR;SLC6A3;GPR68;SH2D1B;AVPR1A;F2RL2;CYSLTR1;CCR5;HTR1F;OPRK1;GPR143;SLC8A3;C3AR1;TGFB2;GRPR;DRD2;INS;ADRB1;NPY;NTSR1;GPR6;GPER1;CCKBR;NPFFR2;PTH1R	8.65E-07
motor behavior	160	59	CAMK2A;VIP;ADORA2A;BDNF;STAC3;CRH;HRH3;OXTR;HRH2;OPRM1;VSX2;EGF;GPR37;SST;RGS6;CASP1;TACR1;SHH;HTR2A;CNR2;RHOA;GSK3B;SLIT3;MANF;ACHE;SLCO3A1;RAC3;ESR2;MAPK1;SIGMAR1;GMFB;TRPC6;GRM1;IGF2;MTOR;PARK7;PTEN;IP6K2;SYNGAP1;PRKN;GRIN2B;IGF1R;CNR1;SLC1A2;NOS2;TRHR;HTR1A;CBLN4;IL10;CDKN2A;CHRNA7;SLC6A3;OPRK1;DRD2;INS;NTSR1;NR4A2;CYP19A1;DRD4	9.80E-07
vision	327	111	CLRN1;ADORA2A;PRPH2;BDNF;KCNV2;SLC16A3;NTRK2;CRH;NYX;CCKAR;HRH3;ARRB1;TNNT2;EDN1;SST;GNAQ;GLRA3;GRK7;EDNRB;CYP1B1;LOX;GRIN2C;DKK1;GRIN1;UNC119;PLIN2;CFTR;SLC1A4;HTR2A;PDE5A;DHRS11;PVALB;RDH12;HSPA5;MYO6;CNR2;PSME1;GSK3B;NDUFAF7;ACHE;CKB;RDH13;ASNA1;ADIPOR1;OPA1;FLT1;PDE6B;OPA3;SLC1A5;PLCB4;GBA;MAPK1;MAPK3;ABCG4;GPHN;APP;NSF;PDC;GRM2;UNC80;GALNS;BSG;SERPINF1;TFPI;NR2C1;VEGFA;NONO;CFH;PPARA;PTEN;NPTN;RGS9;TRPV1;BEST1;MERTK;SLC8A1;NCAM1;SOCS3;CNR1;POC1B;PPEF1;RGS7;RPE65;DDIT4;GPR179;GCGR;PROM1;ELOVL4;PCARE;GUCY2D;NOS2;SLC17A7;ARR3;RGR;CABP4;HTR1A;LRAT;CAPN2;POU4F3;RDH8;GRM6;GNAT1;DRD2;CABP5;INS;GNGT1;NPY;ATOH7;ATP2B2;TMEM145;DRD4	2.06E-06
renal reabsorption	206	70	VIP;REN;DPEP1;SLC28A2;SLC15A1;SLC5A10;SOST;EDN1;EGF;SST;SLC5A2;EDNRB;CNNM2;CLDN19;SLC5A8;SLC13A1;SLC23A1;SLC7A7;SLC7A8;FOLR1;SLC6A8;SLC22A5;AGTRAP;SGK1;SLC9A3R1;SLC5A6;SLC6A19;DPP4;SLC31A1;B2M;SLC5A9;STC2;SLC3A1;CUBN;CLCN5;HIF1A;AGTR2;BAX;SLC19A3;STK39;SLC23A2;AQP1;ARL15;PRKCSH;ACOX1;TGFB1;NR1H4;ARF6;VEGFA;PPARA;IRS2;SLC8A1;VDR;XDH;NFAT5;SLC24A3;PHEX;KL;PPARD;NOS2;TPH1;AQP3;CASR;ESRRG;FGF7;TRPV6;SLC9A2;INS;PTH1R;SLC22A7	2.08E-06
gastric motility	115	36	VIP;PTGER1;CRH;CCKAR;OXTR;HRH2;SLC15A1;OPRM1;EDN1;SST;TACR1;APOA4;SERPINB1;NUCB2;ACHE;COPA;TRPA1;CHRM3;KDR;ADRA2A;NPR3;TRPV1;PDE4D;PRKN;CNR1;TRHR;HTR1A;MC4R;NPPC;TRPC1;SCTR;HTR1F;DRD2;INS;NPY;CCKBR	2.11E-06
peristalsis	93	28	VIP;PTGER1;FFAR2;BDNF;CRH;HRH3;HRH2;OPRM1;EDN1;SST;EDNRB;BMP2;TACR1;CFTR;PDE5A;ACHE;PTGER2;KIT;BMP7;TRPV1;CNR1;GCGR;NOS2;ANO1;TPH1;OPRK1;DRD2;INS	2.11E-06
sexual behavior	148	42	VIP;CRH;CCKAR;OXTR;OPRM1;EGF;VIPR1;SST;ADRA1B;TACR1;SIM1;NHLH2;NTRK1;HTR2A;ESR1;PDE5A;GRN;PRLR;SRD5A1;PTGER2;BAX;ESR2;MAPK1;PGRMC1;TIE1;RNMT;APP;THRA;PTEN;IGF1R;CNR1;HTR1A;MC4R;SLC6A3;AVPR1A;OPRK1;DRD2;INS;NPY;GPER1;MC3R;CYP19A1	2.24E-06
penile erection	138	48	VIP;BDNF;OXTR;NINJ1;HRH2;PTGIS;OPRM1;KCNN3;EDN1;SST;HTR2B;ADORA2B;EDNRB;TACR1;SHH;NTRK1;ADA;HTR2A;HMGCR;PDE5A;RHOA;ACHE;PDE3A;SRD5A1;PARP1;NT5E;DCN;MAPK1;ANXA1;TRPC6;TGFB1;SERPINF1;NR1H4;VEGFA;NR2C2;ADRA1A;CNR1;NOS2;HTR1A;MC4R;NPPC;GDF5;GRPR;DRD2;INS;MC3R;CYP19A1;DRD4	2.57E-06
depressor response	72	26	REN;ADORA2A;BDNF;CRH;HRH2;KCNN3;EDN1;SLC5A2;EDNRB;TACR1;SLC6A2;SLC2A1;TRPA1;CHRM3;PTGER2;AGTR2;VEGFA;TRPV1;CNR1;HTR1A;OPRK1;GPR143;DRD2;INS;ADRB1;NPY	2.80E-06
non-REM sleep	113	36	RAB3A;VIP;REN;ADORA2A;BDNF;CRH;HRH3;EDN1;EGF;SST;HTR2B;CASP1;ADA;HTR2A;TNFRSF1A;NT5E;CSNK1E;APP;TGFB1;GRM2;UBE3A;PER3;IL1R1;CNR1;CRY1;NOS2;HTR1A;IL10;CHRNA7;SLC6A3;SLC6A1;NLGN2;DRD2;INS;NPY;CCKBR	3.05E-06
noradrenaline uptake	59	22	SNCG;RAB3A;ADORA2A;BDNF;DNM1;NTRK2;OPRM1;EDN1;P2RY12;SLC6A2;MYLK;RAB11A;ADRA2A;ENPP2;CNR1;SLC18A2;HTR1A;NPPC;SLC6A3;INS;NPY;AANAT	3.51E-06
baroreflex	148	52	VIP;REN;ADORA2A;BDNF;NTRK2;CRH;HRH3;OXTR;FAS;OPRM1;EDN1;SST;SLC5A2;TACR1;GRIN2C;SLC6A2;HTR2A;ESR1;HMGCR;PDE5A;CTH;SLC2A1;GPR182;PPP1CB;ACHE;LEPR;TRPA1;MSRA;PTGER2;PARP1;AGTR2;ADRA2A;F2RL1;ADAM17;PTPN1;VEGFA;PPARA;CYBB;TRPV1;XDH;CNR1;ADCY5;KL;NOS2;HTR1A;NPPC;AVPR1A;OPRK1;INS;ADRB1;NPY;CYP19A1	3.96E-06

vasoconstriction	523	161	VIP;REN;PGF;PTGER1;PTGIR;ADORA2A;BDNF;WNT5A;TRPM4;CRH;HRH3;OXTR;HRH2;CYGB;PRKCQ;PTGIS;OPRM1;KCNC4;EDN1;P2RY12;EGF;C1QTNF1;SST;GNAQ;ADRA1B;HTR2B;SLC5A2;ADORA2B;EDNRB;TACR1;LOX;NTRK1;SLC6A2;CFTR;ADA;HTR2A;ESR1;LGALS1;SLC9A3R1;DPP4;ALOX12;PDE5A;NAMPT;S1PR2;MB;BET1;TNFRSF1A;ABCC9;ENTPD8;CTH;UTS2R;PRKAA2;ACLY;RGS4;RHOA;TGM2;CBS;MYLK;PDE3A;MYH11;TRPA1;ARG2;HIF1A;PRKCD;CHRM3;FLT1;SMPD2;SGPP1;TNS2;IKBKB;PTGER2;ADRA1D;NT5E;AGTR2;DDAH1;RGS5;ESR2;CACNA1C;MAPK1;NTN1;STK39;PLA2G6;KDR;EMILIN1;F2R;MAPK3;PDGFB;GNA11;THBS1;RNMT;APP;ADRA2A;TRPC6;TGFB1;F2RL1;ARRB2;ITLN1;ADAM17;NDUFA1;ITGB2;PRKAA1;GJA1;STAT3;NR1H4;KLHDC2;ATP5J;VEGFA;TBXAS1;NPR3;ATP2B1;CYBB;PPARA;MYL12B;CYR61;NFATC3;TRPV1;LRP1;SGMS2;FN1;PRKCE;ADRA2B;SLC8A1;ADRA1A;F13A1;UCP3;VDR;XDH;CNR1;HK2;LPAR2;PRKCB;KL;PPARD;NOS2;ANO1;HTR1A;ADORA3;CNGA2;IL10;NPPC;EPHB6;TRPC1;CASR;SLC6A3;GPR68;AVPR1A;CYSLTR1;CCR5;LPL;HTR1F;OSTN;TGFB2;DRD2;INS;ADRB1;NPY;GPER1;ABCG1;MTNR1B;CYP8B1;DRD4	4.01E-06
HCO(3)(-) transport	164	54	VIP;KCNJ10;PTGER1;FFAR2;CRH;CCKAR;CA4;HRH2;EDN1;EGF;FFAR3;SST;HTR2B;ADORA2B;EDNRB;TAS1R3;SLC4A8;SLC5A8;PDZK1;CFTR;ADA;ESR1;SLC9A3R1;DPP4;PDE5A;AHCYL1;CTH;OGDH;ACHE;PLA2G4A;CHRM3;ESR2;MAPK1;STK39;SLC26A3;BEST2;SLC4A2;AQP1;F2RL1;SLC9A8;NR1H4;TRPV1;BEST1;PRKCE;SLC8A1;HRAS;NOS2;ANO1;ENTPD2;CASR;SCTR;SLC9A2;INS;NPY	4.36E-06
cholinergic synaptic transmission	123	41	VIP;ADORA2A;BDNF;TRPM4;HRH3;CHRNA1;ESM1;OPRM1;EDN1;SST;EDNRB;TACR1;UNC13A;TULP4;ESR1;CNR2;GSK3B;ACHE;PREP;CHRM3;SIGMAR1;NEU1;APP;TGFB1;GRM1;STAT3;HTR6;PPARA;CYR61;CNR1;HTR1A;ADORA3;CHRNA7;ENTPD2;OPRK1;DRD2;INS;ADRB1;NPY;SLC18A3;DIRAS1	4.63E-06
menopause	85	27	REN;BDNF;ENPEP;OXTR;SOST;OPRM1;EDN1;CYP1B1;DKK1;MTHFR;ESR1;ALOX12;APOA1;PVALB;SRD5A2;CXCR4;ESR2;SEMA3A;VDR;F7;SOCS3;PPARD;IL10;LPL;SLC9A2;INS;CYP19A1	4.82E-06
bronchoconstriction	169	53	VIP;REN;PTGER1;ADORA2A;BDNF;ALOX5AP;HRH3;HRH2;ARRB1;OPRM1;EDN1;SST;ADORA2B;EDNRB;TACR1;HTR2A;EPX;SERPINB1;PDE5A;APOA1;CNR2;RGS4;RHOA;MYLK;ITGA4;ACHE;TRPA1;NCF4;PLA2G4A;CHRM3;PTGER2;NT5E;NEU1;F2R;F2RL1;TGFB1;PTPRJ;PGGT1B;VEGFA;TBXAS1;TRPV1;XDH;CNR1;NOS2;ANO1;HTR1A;ADORA3;CTRL;NPPC;TRPC1;CYSLTR1;INS;NPY	5.28E-06
Ca++ export	1048	354	CRHBP;CSRP3;CAMK2A;RAB3A;VIP;RGS18;PGF;DEF6;PTGER1;FFAR2;ADORA2A;LCK;BDNF;STAC3;CALCR;WNT5A;MAG;NTRK2;CD79A;TRPM4;CRH;CCKAR;HRH3;OXTR;IL4I1;HRH2;CHRNA1;FAS;OGN;PRKCQ;DMTN;SOST;OPRM1;SAA1;LTB4R;CASQ2;TLR2;EDN1;TWIST1;PIP4K2C;LCP2;P2RY12;EGF;FFAR3;CALHM1;C1QTNF1;SST;GPRC5B;GNAQ;ADRA1B;HTR2B;SLC5A2;ADORA2B;EDNRB;CERS6;BMP2;TMEM38A;TACR1;P2RY13;NMUR2;HHATL;CACNA1S;UNC13A;NTRK1;GPX1;NCF1;RCN3;LGALS9;CFTR;LYN;HTR2A;ESR1;LGALS1;RTN1;KRAS;CORO1A;ANO5;PDE5A;HYOU1;S1PR2;PTPRC;AHCYL1;GPD2;DNAJB11;MFN2;TNFRSF1A;PVALB;ERO1A;CALU;B2M;PTRH2;HSPA5;CS;UTS2R;CNR2;RSPO1;RGS4;LETM1;RHOA;GSK3B;TGM2;RCN1;MRVI1;SLC2A1;HSPA9;MCAM;TMCO1;PECAM1;PPA1;MORN4;CBS;CCDC47;BCAP31;MYLK;HSP90AA1;GRAP2;DBI;HMGB1;GRB2;LEPR;MAPK9;STIP1;RAP1A;TRPA1;RAP1B;CASP8;CXCR4;PANX3;RAB27A;CLN8;JAK3;PLCH1;PRKCD;PLA2G4A;ZAP70;GNA13;CHRM3;ATF4;FLT1;SRI;SNAPIN;MAP2K2;TFEB;PDE4B;PON2;PTGER2;GLRX;BTK;ATP1A1;CDC42;PPIB;PARP1;PLPP1;PLCB4;GBA;SPRY4;AGTR2;YWHAZ;FAM3A;TMBIM4;BAX;GSR;RGS5;CA8;INHBA;CACNA1C;RASGRP3;GNB1;SELENOK;CSNK2B;EIF4A3;MAPK1;PPID;SLC35G1;EFHD2;NUCB1;SIGMAR1;AHCYL2;AIP;NTN1;RGS1;PLA2G6;OMP;KDR;NDUFS3;CALR;CTSK;FUNDC1;AMFR;JUN;F2R;ERP44;MAPK3;STOML2;TNFSF13B;SGCD;MICU1;NQO1;RGN;NAPA;OSBP;PSEN2;RAN;GUSB;GNA11;INPP5D;ERN1;ALK;APP;ABCA1;ANO10;PRKCSH;ADRA2A;ATP7A;PPP2R5A;ANXA1;TRPC6;SELENOT;TGFB1;F2RL1;CCR2;PTPN11;FIS1;BSG;ORMDL3;LAMC1;E2F1;CLIC2;ZDHHC6;GRM1;EIF2S1;ITGB2;TXN;TLN1;GJA1;HOMER2;ITPKB;STAT3;MTOR;HSP90B1;NOA1;PTPN1;HTR6;RPL23;ARF6;VEGFA;PNPLA8;CFLAR;HOMER1;SV2A;SEPN1;FKBP1A;SEC61A1;CSK;ATP2B1;CYBB;NFATC3;GNG7;PTEN;GPI;AHNAK;AKAP6;LRRK2;NRIP1;TLR1;PIP5K1A;TRPV1;GRINA;ITPRID2;BEST1;ADCY6;PDE4D;VDAC1;PLCE1;FN1;PRKCE;PRICKLE1;MCOLN1;SLC8A1;ADRA1A;UCP3;VDR;F7;PTK2;IGF1R;GM2A;NCAM1;NR4A1;XDH;CNR1;SLC24A3;PARP2;TENM2;RGS7;ANPEP;MCL1;PRICKLE2;RRM2;HRAS;CELSR3;GCGR;SCN5A;CACNA1D;PRKCB;SLC24A4;NOS2;ANO1;CRYAB;GABBR1;TRHR;KCNIP2;HTR1A;SLC8A2;ADORA3;MC4R;IL10;NPPC;CAPN2;CHRNA7;FSHR;GDF11;TRPC1;CASR;GPR68;AVPR1A;F2RL2;TRPV6;CYSLTR1;NCS1;CCR5;OPRK1;SLC8A3;C3AR1;OSTN;GRPR;CACNB3;DRD2;S100A4;WNT4;DHRS7C;INS;ADRB1;NPY;ANKRD1;NTSR1;LIM2;GPER1;CCKBR;MC3R;BIN1;MPP4;ATP2B2;PTH1R;DRD4	5.53E-06
action potential	462	148	CSRP3;VIP;REN;MRC1;KCNJ10;SCN4B;BDNF;KCNV2;SCN1B;KCNH1;TRPM4;CRH;OXTR;KCNK18;RAB11B;FAS;CAV3;OPRM1;KCNC4;KCNN3;TNNT2;EDN1;CALHM1;KCNMB2;SST;KCNB2;KCNJ11;SLC5A2;TACR1;GRIN2C;KCNQ3;CACNA1S;SHH;DSG2;CFTR;ADA;HTR2A;ESR1;GCK;SGK1;PVALB;CTH;GSK3B;ACHE;HMGB1;RAP1A;TRPA1;PLA2G4A;CHRM3;ATP23;SMPD2;AGTR2;BAX;CACNA1C;INHBA;MAPK1;SIGMAR1;NEU1;PLA2G6;ZFPM2;OMP;KDR;RPS3;RPS6;JUN;F2R;JUP;MAPK3;DTNBP1;PSEN2;CLCN3;APP;SNX9;BVES;TRPC6;CYP2J2;TGFB1;F2RL1;CCR2;GALNS;GRM1;VIPR2;GJA1;HSPA8;NOTCH1;VEGFA;KIT;SV2A;FKBP1A;EXOC5;ATP2B1;NKX25;VAMP3;GATA4;PTEN;ERBB4;FURIN;TRPV1;ADCY6;FN1;PRKCE;SEMA3A;SLC8A1;ADRA1A;ATP1A3;NCAM1;XDH;CNR1;LGI1;SCN5A;CACNA1D;ST8SIA2;KL;DPP6;KCNC2;MARCH7;NOS2;ANO1;SCN8A;SLC17A7;KCND1;NAV1;KCNIP2;HTR1A;MC4R;NPPC;CHRNA7;CACNA2D3;FSHR;CASR;SLC6A3;KCNC1;SLC6A1;AVPR1A;DLG4;OPRK1;KCNK16;GNAT1;ERG;DRD2;INS;ADRB1;NPY;NTSR1;SLC45A2;KCNAB1;GLRA2;DRD4	5.88E-06

membrane polarization	272	98	VIP;REN;PGF;MRC1;SCN4B;BDNF;KCNV2;KCNH1;TRPM4;CRH;CAV3;KCNC4;KCNN3;CASQ2;EDN1;PIP4K2C;CALHM1;KCNMB2;SST;GNAQ;KCNB2;ADRA1B;KCNJ11;SLC5A2;CACNA1S;SHH;CDH17;CFTR;ESR1;SGK1;DPP4;ALOX12;KCNE4;RHOA;GSK3B;PECAM1;ACHE;MYH6;TRPA1;PLA2G4A;CHRM3;TFEB;MARK2;CDC42;EZR;AGTR2;RGS5;ESR2;CACNA1C;NEU1;OMP;KDR;RPS3;RANGAP1;LGALS3;RAB11A;RAN;CLCN3;AQP1;APP;THRA;CYP2J2;TGFB1;DNM1L;GJA1;STAT3;NOTCH1;VEGFA;CYBB;PTEN;SIRT2;TRPV1;ADCY6;PRKCE;SLC8A1;UCP3;XDH;KIF17;SCN5A;CACNA1D;DPP6;ANO1;CRYAB;KCNIP2;NPPC;TRPC1;CASR;SLC6A3;KCNC1;CCR5;KCNJ6;KCNK16;ERG;INS;ADRB1;NPY;IRX4;CDH1	6.80E-06
neuronal activity	669	214	CAMK2A;VIP;REN;KCNJ10;PTGER1;SCN4B;ADORA2A;OPCML;BDNF;KCNH1;NTRK2;NDUFA4;CRH;FABP7;CCKAR;HRH3;OXTR;HRH2;KCNK13;SLC5A11;OPRM1;KCNN3;EDN1;IGSF9B;P2RY12;EGF;VIPR1;CALHM1;SST;GNAQ;HTR2B;PTN;ADORA2B;TACR1;GRIN2C;KCNQ3;NFATC4;DKK1;NPAS4;GRIN1;UNC119;UNC13A;SHH;NTRK1;SLC4A8;MTHFR;CFTR;HTR2A;ESR1;GCK;PDE5A;NAMPT;TNFRSF1A;PVALB;DUSP6;HSPA5;CTH;SCG2;CNR2;RHOA;GSK3B;SLC2A1;PACSIN1;CBS;DBI;ACHE;SPI1;HMGB1;LEPR;PRLR;TRPA1;CPT1A;CXCR4;MSRA;HSPD1;ADIPOR1;SYP;HIF1A;ATF4;RABGEF1;CDC42;ADRA1D;ATAD1;AGTR2;BAX;PTPRE;ESR2;CACNA1C;MAPK1;CALHM2;SIGMAR1;NEU1;PCDH10;SDCBP;NTN1;OMP;RPS3;DAO;SEMA3F;RPS6;SP4;F2R;MAPK3;EFNA2;RNMT;APP;AQP1;LXN;ADRA2A;WWC1;DNM3;EIF4EBP1;TGFB1;GRM2;RAB35;UNC80;DNAJA3;GRM1;NRP2;UBE3A;TXN;MEF2A;GJA1;RPS6KB1;NR1H4;NOTCH1;MTOR;HTR6;ARF6;VEGFA;HOMER1;PARK7;SV2A;TCF7L2;ADCY3;CYBB;PPARA;PTEN;NPTN;SEMA4D;NRF1;ERBB4;RBFOX1;TRPV1;ADCY6;TNIK;TFAP2C;SYNGAP1;PRKN;PRKCE;SLC8A1;ADRA1A;GRIN2B;CTTNBP2;VDR;PTK2;ADCY1;IGF1R;NCAM1;NR4A1;KIRREL3;CNR1;APBA1;CRY1;TBR1;NELL2;GLUL;ADCY5;SLC1A2;CACNA1D;PRKCB;OTX1;KL;PPARD;APBA2;NOS2;ELAVL2;SCN8A;GABBR1;KCND1;CABP4;NCAM2;HTR1A;ADORA3;MC4R;CNGA2;IL10;NPPC;TPH1;CHRNA7;CASR;SLC6A3;KCNC1;SLC6A1;AVPR1A;GRM6;EFNB3;NCS1;HTR1F;DLG4;OPRK1;FGF14;DBH;DRD2;INS;FGFBP3;ADRB1;NPY;NTSR1;GPER1;CCKBR;MTNR1B;CYP19A1;DRD4	7.14E-06
migrating myoelectric complexe	42	11	VIP;CRH;CCKAR;OXTR;SST;HTR2A;CHRM3;HTR1A;INS;NPY;CCKBR	7.23E-06
social behavior	217	75	VIP;PTGER1;ADORA2A;BDNF;FGF17;NTRK2;CRH;CCKAR;HRH3;OXTR;MRAS;OPRM1;EGF;SST;NLGN4X;PRSS12;GRIN2C;GRIA3;NPAS4;GRIN1;NCF1;SCIN;HTR2A;ESR1;GDI1;PVALB;MYC;RGS4;SYN2;GSK3B;TGM2;PPA1;GRN;TRPA1;GLUD1;NT5E;BAX;ESR2;CACNA1C;MAPK1;PCDH10;PIK3CB;MAPK3;GADD45B;ERN1;APP;UBE3A;MTOR;PTEN;CADM1;ERBB4;EHMT2;CLIP2;SYNGAP1;FN1;DLGAP2;GRIN2B;VDR;CNR1;SARM1;CACNA1D;APBA2;HTR1A;TSHR;SLC6A3;AVPR1A;CCR5;OPRK1;NLGN2;GRPR;DRD2;INS;NPY;GPER1;CYP19A1	7.35E-06
maternal behavior	70	27	CRHBP;ADORA2A;BDNF;STMN1;CRH;CCKAR;OXTR;OPRM1;HTR2A;ESR1;B2M;PRLR;MBD2;MEST;JUN;IGF2;NR2C2;CREBRF;CNR1;SLC6A3;AVPR1A;OPRK1;DBH;DRD2;NPY;GPR101;DRD4	7.74E-06
membrane hyperpolarization	504	170	VIP;KCNJ10;SCN4B;FFAR2;ADORA2A;BDNF;KCNV2;KCNH1;TRPM4;CRH;HRH3;OXTR;IL4I1;HRH2;KCNK18;FAS;PRKCQ;OPRM1;KCNN3;EDN1;FBXL13;PIP4K2C;EGF;CALHM1;KCNMB2;SST;KCNB2;KCNJ11;ADORA2B;EDNRB;CASP1;TACR1;KCNQ3;NTRK1;APOA4;SLC5A8;CFTR;ADA;HTR2A;GCK;SGK1;PDE5A;APOA1;PVALB;ABCC9;SLC25A27;PTRH2;CTH;ACLY;CNR2;GSK3B;TGM2;NDUFAF7;MYLK;KCNJ13;ACHE;LEPR;KCNG1;TRPA1;CASP8;CXCR4;NDUFAF1;CLIC1;HIF1A;CHRM3;FLT1;SMPD2;IKBKB;PTGER2;FSTL1;ATP1A1;ATP5MF;NT5E;AGTR2;BAX;GSR;ESR2;MAPK1;PPID;SIGMAR1;NEU1;PDK2;PIK3CB;RPS3;SLC26A3;HNF4A;HSD17B10;GADD45GIP1;ERN1;APP;ADRA2A;BVES;TRPC6;TGFB1;F2RL1;GRM2;FIS1;GSTP1;GRM1;VIPR2;ATP5H;PRKAA1;GJA1;NOTCH1;MTOR;NOA1;VEGFA;HOMER1;PARK7;NPR3;ATP2B1;PPARA;MFN1;VTN;IL1R1;RGS9;STARD3;TRPV1;VDAC1;FN1;PRKCE;SEMA3A;SLC8A1;GRIK2;UCP3;PTK2;ENPP2;KCTD7;IGF1R;XDH;CNR1;IRS1;MCL1;HK2;LPAR2;SLC1A2;SCN5A;CACNA1D;PPARD;DPP6;NOS2;ANO1;SCN8A;HTR1A;ADORA3;MC4R;IL10;NPPC;ENTPD2;TRPC1;CASR;SLC6A3;KCNC1;GRM6;TRPV6;CSNK2A1;LPL;DLG4;OPRK1;KCNJ6;KCNK16;GNAT1;DRD2;INS;ADRB1;NPY;NTSR1;ABCG1;KCNAB1;DRD4	8.27E-06
turning behavior	47	22	EN2;ADORA2A;BDNF;MAG;HRH2;EDN1;SST;HTR2B;PTN;VIM;MYLK;MANF;ACHE;MAPK1;NTN1;VEGFA;CNR1;TPH1;SLC6A3;DRD2;NPY;NTSR1	9.08E-06
pituitary gland function	131	36	VIP;REN;BDNF;CALCR;CRH;OPRM1;EGF;SST;FGF4;SHH;ESR1;ALOX12;SCG2;ACHE;RAP1A;CXCR4;RIPK1;FST;INHBA;SOX18;TGFB1;IGF2;VEGFA;PLAG1;IGF1R;CNR1;OTX1;TRHR;C3AR1;PTH2R;DRD2;INS;NPY;GPER1;MC3R;AANAT	9.73E-06
licking	92	36	PTGER1;ADORA2A;CRH;OXTR;HRH2;OPRM1;EDN1;SST;TACR1;TAS1R3;DKK1;NTRK1;HTR2A;ESR1;TNFRSF1A;TRPA1;ESR2;MAPK1;SIGMAR1;THOP1;GRM2;ARRB2;GRM1;TRPV1;GRIN2B;SPX;EPHB1;NOS2;HTR1A;SLC6A3;AVPR1A;DRD2;INS;ADRB1;NPY;NTSR1	1.08E-05
action potential duration	217	77	VIP;REN;MRC1;SCN4B;BDNF;SCN1B;TRPM4;HRH2;FAS;CAV3;KCNC4;KCNN3;EDN1;SST;GNAQ;KCNB2;KCNJ11;TACR1;PRF1;SHH;CFTR;HTR2A;LGALS1;ABCC9;CTH;CNR2;GSK3B;HSPB1;ACHE;HMGB1;BAG3;CHRM3;SRI;ITGAM;AGTR2;RGS5;CACNA1C;SIGMAR1;NEU1;AIP;KDR;NET1;RAN;CLCN3;APP;TGFB1;RPL14;GJA1;HSPA8;NOTCH1;VEGFA;NKX2-5;ADCY6;SEMA3A;SLC8A1;ADRA1A;ENPP2;XDH;CNR1;SCN5A;CACNA1D;DPP6;KCNC2;NOS2;SCN8A;CRYAB;KCNIP2;HTR1A;NPPC;CASR;SLC6A3;KCNC1;INS;ADRB1;NPY;KCNAB1;BIN1	1.13E-05
inotropism	239	89	VIP;REN;PTGER1;ADORA2A;CALCR;TRPM4;CRH;HRH3;HRH2;MYH7;ARRB1;EDN1;P2RY12;EGF;VIPR1;GUCY1A1;SST;GNAQ;ADRA1B;HTR2B;ADORA2B;EDNRB;TACR1;CFTR;ADA;HTR2A;ODC1;DPP4;PDE5A;TNFRSF1A;PVALB;EIF4E;CNR2;HAX1;RHOA;SLC2A1;GPR182;GLRX3;MYLK;ACHE;HMGB1;TRPA1;CXCR4;ARG2;HIF1A;PRKCD;CHRM3;ADRA1D;AGTR2;CACNA1C;MAPK1;NEU1;PLA2G6;F2R;MAPK3;NQO1;GNA11;ANXA1;ARRB2;VIPR2;BIRC2;GJA1;STAT3;VEGFA;TXNIP;MYL12B;CYBB;PTEN;VTN;TRPV1;PRKCE;ADRA1A;IGF1R;CNR1;CCNA2;GCGR;PPARD;NOS2;ADORA3;NPPC;CASR;AVPR1A;OPRK1;DBH;INS;ADRB1;NPY;GPER1;PTH1R	1.32E-05
